# Identification of the first peptide inhibitor of UBE2C enzymatic activity: insights from metadynamics-guided folding and binding studies

**DOI:** 10.1080/14756366.2025.2605383

**Published:** 2026-01-06

**Authors:** Luciano Pirone, Bianca Fiorillo, Annarita Del Gatto, Rita Russo, Alessandra Guarracino, Chiara Cassiano, Laura Zaccaro, Federica Moraca, Emilia Pedone, Bruno Catalanotti

**Affiliations:** ^a^ Institute of Biostructures and Bioimaging, Naples, Italy; ^b^ Department of Pharmacy, University of Naples “Federico II”, Naples, Italy

**Keywords:** Ubiquitin conjugation enzymes, UBE2C, cancer, metadynamics, peptide-protein interaction

## Abstract

UBE2C (also known as UbcH10) is a ubiquitin-conjugating enzyme essential for mitotic progression and a potential therapeutic target in cancer. Here, we report a structure-based design and characterisation of peptides derived from a natural interacting partner (U1) aimed at modulating UBE2C activity. Biophysical and biochemical assays identified peptide **5** as a lead compound, capable of binding UBE2C with micromolar affinity and inhibiting the formation of the UBE2C-Ub thioester complex. Enhanced sampling molecular dynamics simulations revealed that peptide folding landscapes are correlated with activity, with active peptides sampling transient β-sheet conformations compatible with binding. To the best of our knowledge, this is the first report of a peptide inhibitor of UBE2C enzymatic activity.

## GRAPHICAL ABSTRACT

**Figure:**
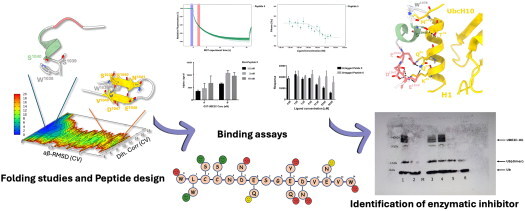

## Introduction

**Figure 1.**
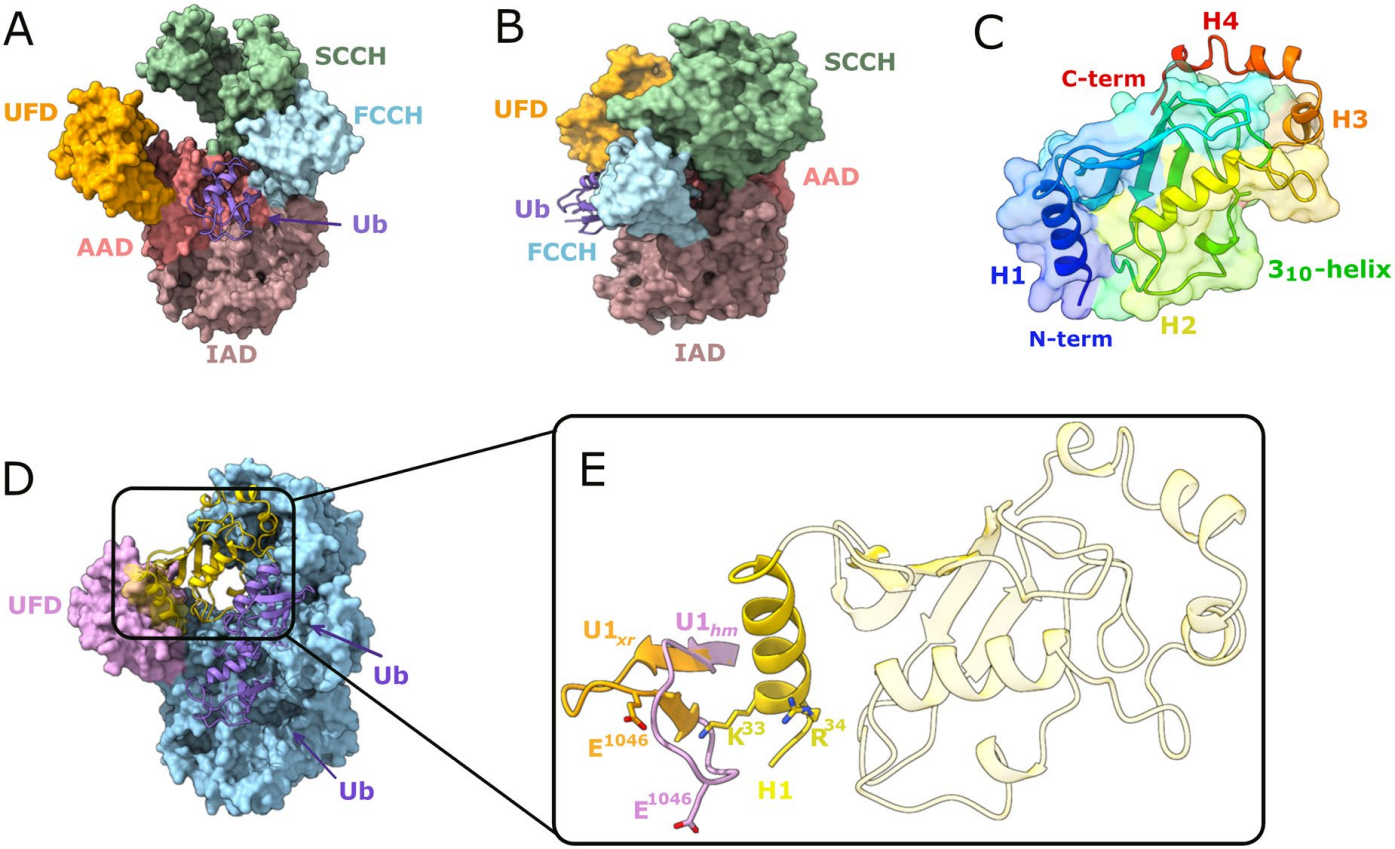
Structural domains of *h*UBA1 and *h*UbcH10. (A) Front and (B) Lateral views of the *h*UbA1/Ub complex (PDB ID: 6DC6), Ubiquitin (Ub) is shown as violet cartoon, while *h*UbA1 is shown as surface coloured by structural domains; (C) The overall fold of *h*UbcH10 with the H1-H4 α-helices, 3_10_-helix, and four β-strands. The area surrounded by a transparent surface indicates the UBC domain, while the active site residue (C^114^) is depicted as green stick. (D) Homology model of *h*UbA1/2Ub/UbcH10 quaternary complex, with a focus on the fragment U1 folding of the homology model (U1*_hm_*) (plum cartoon/surface) interacting with the H1 helix of *h*UbcH10 (yellow cartoon/surface); (E) Superposition between the β-sheet conformation of U1 peptide (U1_xr_) retrieved from the X-Ray structure (PDB ID: 6DC6) (orange cartoon) and the coil/turn motif of U1 (U1_hm_) obtained from the homology model of the complex *h*UbA1/UbcH10/Ub^31^ (plum cartoon).

Ubiquitin conjugation enzymes, also known as E2 enzymes, are central to the ubiquitin-proteasome system (UPS), which is a critical pathway for protein degradation in eukaryotic cells. The process of ubiquitination involves a cascade of enzymatic reactions, in which ubiquitin is first activated by ubiquitin-activating (E1) enzymes (Figure 1(A,B)), then transferred to E2 enzymes, and finally conjugated to substrate proteins by E3 ligases^1^. This post-translational modification plays a pivotal role in regulating various cellular processes, including cell cycle progression, DNA repair, apoptosis, and protein quality control^2^.

E2 enzymes determine the specificity and type of ubiquitin chain formed, which in turn dictates the fate of the target proteins, whether they are marked for proteasomal degradation or modulated for other cellular functions^3^. The human genome encodes approximately 35 distinct E2 enzymes, enabling a broad spectrum of functional outcomes, making these enzymes key regulators in various physiological processes^4^. Importantly, dysfunction in the ubiquitin-conjugation system has been implicated in a variety of diseases, including cancer, where abnormal regulation of ubiquitination leads to tumour progression and resistance to therapies^5^.

Despite the crucial roles that Ubc family members play in regulating protein degradation and cell cycle processes, inhibitors for only a few Ubcs have been identified.

In this context, peptide-based inhibitors have emerged as a promising therapeutic modality due to their high specificity and potency against a wide range of protein targets. These molecules offer several advantages, including the ability to target protein-protein interactions that are often challenging for small molecules, as well as reduced off-target effects^6–9^. Computational approaches, including molecular docking and molecular dynamics simulations, coupled with free energy evaluation methods have significantly accelerated the discovery of novel inhibitors by providing detailed insights into binding mechanisms and protein-ligand interactions^10–12^.

The development of Ubc inhibitors has become a key focus in therapeutic research due to their crucial role. Recent research has identified significant advancements in the development of inhibitors targeting ubiquitination and SUMOylation pathways, both critical for cellular regulation and implicated in diseases such as cancer. Ubiquitination inhibitors primarily focus on enzymes like Ubc13^13–17^, Cdc34^18^, Rad6^19^^,^^20^, and UBE2g2^21^, which play pivotal roles in protein signalling and degradation.

In contrast to ubiquitination, SUMOylation inhibitors focus on Ubc9, the sole E2 enzyme in this pathway. Small-molecule allosteric inhibitors of Ubc9 have emerged as promising candidates, blocking SUMO conjugation and disrupting cancer-related SUMOylation pathways^22–25^. These discoveries highlight a diverse arsenal of inhibitors targeting distinct nodes within ubiquitination and SUMOylation pathways. Nevertheless, the research was focused only on a few among the 35 known members of the family.

Among the E2s, a significant role in tumour progression and resistance to many pharmacological treatments is played by UBE2C (also known as UbcH10) (Figure 1(C)). The E2 enzyme UBE2C plays a pivotal role in cell cycle regulation through its involvement in the UPS, which targets key regulatory proteins for degradation. UBE2C, as an E2 enzyme, works closely with the anaphase-promoting complex/cyclosome (APC/C) during the metaphase-to-anaphase transition, facilitating the ubiquitination of substrates such as cyclin B1. This activity is crucial for maintaining proper cell cycle progression and ensuring genomic stability^26^.

Overexpression of UBE2C has been implicated in several cancers, where its dysregulation promotes tumorigenesis by enhancing cell proliferation and inhibiting apoptosis^27^. For example, UBE2C is highly expressed in various cancers, including gastric, lung, and breast cancers, and is correlated with tumour aggressiveness and poor prognosis^21^^,^^24–27^. Moreover, studies have shown that silencing UBE2C leads to decreased tumour cell proliferation and increased apoptosis, highlighting its potential as a therapeutic target^28^^,^^29^. Thus, UBE2C is not only essential for normal cell cycle regulation but also represents a key player in oncogenesis, making it a promising biomarker and therapeutic target in cancer treatment. The 3D structure of E2 enzymes, including UBE2C, typically consists of a compact α + β fold protein, featuring a central 4-stranded antiparallel β-sheet flanked by four α-helices, and a highly conserved ubiquitin-conjugating catalytic (UBC) domain spanning nearly the entire 150–200 amino acid polypeptide chain (Figure 1(C)). This domain is crucial for their function in catalysing the transfer of ubiquitin to substrates, thanks to the C^114^ residue of the active site. Structural studies reveal that E2 enzymes, despite sharing a common catalytic core, show small structural differences in loop regions that contribute to their functional diversity. For instance, UBE2C has unique insertion sequences that may affect its interactions with specific E3 ligases, giving it substrate specificity^2^. Unfortunately, although several experimental structures of the E1 enzyme Uba1 from yeast in complex with the E2s UBC4 or CDC34 have been published, only one experimental structure of human Uba1 in complex with ubiquitin alone (*h*UbA1/Ub) has been released in 2018^30^, but no experimental structure of the *h*UbA1/Ub/*h*UBE2C ternary complex is available in the Protein Data Bank (PDB). Nonetheless, valuable structural insights of the molecular basis of the interaction between *h*UbA1 and *h*UBE2C, have been proposed in 2015 by our research group through a computational model constructed using an experimentally guided strategy that combined homology modelling, protein-protein docking and refinement using Steered Molecular Dynamics (SMD) simulations (Figure 1(D)), with a very good overlap of *h*UbA1 with the X-Ray model (RMSD = 1.88 Å). To experimentally validate the E1/E2 interaction predicted by our previous computational studies, we identified two peptides derived from distinct domains of human UBA1 (Supplementary Material, Figure S1A): the **U1** peptide (1038-LCCNDESGEDVEV-1050) from the UFD domain (Supplementary Material, Figure S1B), and the **L2** peptide (615-TESYSSSQDPPEK-627) from the AAD/SCCH domain (Supplementary Material, Figure S1C), both of which were found to interfere with E1/E2 complex assembly^31^. Notably, in our predicted model, the **U1** peptide has been identified as the most active one with a binding capability to the H1 helix of UbcH10 of K_d_=10 µM. Furthermore, our computational homology model predicted an unfolded β-turn conformation (U1*_hm_*) (Figure 1(E)), which contrasts with the β-sheet conformation later observed in the crystallographic structure (U1*_xr_*) (PDB ID: 6DC6)^30^ (Figure 1(E)).

Taking advantage of our previous findings^31^, we now present a multidisciplinary strategy that integrates biochemistry, molecular biology, peptide synthesis, and computational modelling to design, synthesise, and biologically evaluate a series of U1-derived peptides. This effort led to the identification of the first functional inhibitor of UBE2C with improved physico-chemical properties with respect to the parent **U1** peptide. Given the pivotal role of UBE2C in cell cycle progression and tumorigenesis, the discovery of selective inhibitors represents a critical step towards the development of novel anticancer therapeutics.

## Materials and Methods

### Peptide synthesis reagents

Polypropylene reaction vessels and sintered polyethylene frits were supplied by Biotage AB (Uppsala, Sweden), NovaSyn TGR resin, 2-(1H-benzotriazole-1-yl)-1,1,3,3-tetramethyluronium hexafluorophosphate (HBTU), cyano-hydroxyimino-acetic acid ethyl ester (Oxyma), and all amino acids were from Novabiochem-Merck (Nottingham, UK). N,N-diisopropylethylamine (DIPEA), piperidine, trifluoroacetic acid (TFA), Kaiser test, and scavengers were purchased from Sigma–Aldrich (Milan, Italy). N,N-dimethylformamide (DMF) was purchased from CARLO ERBA Reagents (Milan, Italy). Acetonitrile (ACN), dichloromethane (DCM), and diethyl ether were purchased from VWR International (Milan, Italy). All aqueous solutions were prepared by using water obtained from an Arium^®^ Pro Ultrapure Water System (Sartorius, conductivity 0.055 µS/cm (≅18.2 MΩ * cm); TOC content < 5 ppb; Bacteria < 0.001 CFU/mL; No particles > 0.22 μm)

### Peptides synthesis

Peptides listed in Table 1 were obtained by Fmoc solid-phase strategy. Such peptides were manually synthesised by the Fmoc solid-phase strategy (0.1 mmol). The syntheses were carried out on a Novasyn TGA resin (substitution 0.25 mmol g^− 1^) employing all standard amino acids and by using polypropylene reaction vessels fitted with a sintered polyethylene filter. The first amino acid was bound to the support by treating with Fmoc-Val − OH or Fmoc-Trp(Boc)-OH (5 equiv)/MSNT (5 equiv)/MeIm (3.75 equiv) in DCM for 3 h. Coupling reactions were performed by using 10 equiv of Fmoc-protected amino acids activated *in situ* with HBTU (9.8 equiv)/Oxyma (9.8 equiv)/DIPEA (20 equiv) in DMF for 1h. The coupling efficiency was assessed by the qualitative Kaiser test. The Fmoc protecting group was removed by treatment with 30% piperidine in DMF (3 × 10 min). All peptides were cleaved off the resin by treatment with a mixture of TFA/H_2_O/TIS (triisopropylsilane) (95:2.5:2.5 *v/v/v*) for 3h at room temperature. The resins were filtered, and the crude peptides were precipitated with diethyl ether, dissolved in a H_2_O/CH_3_CN (2:1 *v/v*) solution, and lyophilised. The peptides were purified by preparative RP-HPLC on an Agilent Technologies PrepStar equipped with a G9309A 325 Uv-vis Dual Wavelength Detector, using an Aeris Peptide XB-C18 column (250 × 21.2 mm; 5 μm; 100 Å) and a linear gradient of H_2_O (0.1% TFA)/CH_3_CN (0.1% TFA) from 5 to 50% of CH_3_CN (0.1% TFA) in 40 min at flow rate of 20 ml/min. The collected fractions containing the peptides were lyophilised. The identity and purity of peptides were assessed by an LC/MS AGILENT single quadrupole (Agilent 1260 Infinity II LC System) equipped with a diode array detector combined with an electrospray ion source and a quadrupole mass analyser using an Aeris peptide XB-C18 (100 × 4.6 mm; 3.6 μm; 100 Å) and a linear gradient of H_2_O (0.05% TFA)/CH_3_CN (0.05% TFA) from 5 to 70% of CH_3_CN (0.05% TFA) in 30 min at flow rate of 0.8 ml/min.

**Table 1.** Sequences of the designed U1-derived peptides.

Peptide	Sequence	Design phase
U1 (1)	1038-LCCNDESGEDVEV-1050	*lead*
2	1038-L**SS**NDESGEDVEV-1050	*ii*
3	1037-**W**L**SS**NDESGEDVEV-1050	*ii + iii*
4	1038-L**SS**NDESGEDVEV**W**-1051	*ii + iii*
5	1038-**WSS**NDESGEDVEV-1050	*ii + iii*
6	1038-L**SS**NDESG**Y**DVEV-1050	*ii + iii + iv*
7	1038-L**SS**NDESG**Q**DVEV-1050	*ii + iv*
8	1038-L**SS**NDESGEDV**N**V-1050	*ii + iv*
9	1037-**W**L**SS**N**N**ESGEDVEV-1050	*ii + iii + iv*
10	1037-**W**L**SS**ND**Q**SGEDVEV-1050	*ii + iii + iv*
11	1037-**W**L**SS**NDESGE**N**VEV-1050	*ii + iii + iv*

Amino acids highlighted in bold represent the mutated or added residues.

### Recombinant UBE2C production

UBE2C was produced in *E. coli* strain BL21(DE3)GOLD (Invitrogen). UBE2C expression was carried out growing cells at 37◦C and the protein expression was induced by the addition of 1 mM isopropyl β-D-1-thiogalactopyranoside (IPTG). The protein induction was protracted at 22◦C for 16 h. The cells were harvested and the proteins were isolated by sonicating cell pellets resuspended in 30 ml PBS1X in the presence of an EDTA free protease inhibitor cocktail (Roche Diagnostics). The crude cell extract was cleared by centrifugation at 18000 rpm and the supernatant was loaded onto a 5 ml GST-trap column connected to AKTA pure system (Cytiva) equilibrated with binding buffer PBS1X. After washing with ten volumes of binding buffer, a single elution step was performed with 50 mM TrisHCl, 10 mM reduced glutathione. The fractions containing GST-UBE2C were pooled and extensively dialysed against PBS1X at 4 °C. UBE2C was separated from GST tag by proteolytic digestion with Thrombin protease (Sigma Aldrich), using a molar ratio of 1:100 and incubating the sample for 16 h at 4 °C. A second step of GST trap purification was carried out to separate UBE2C from GST. Finally, a further purification step was performed by a size exclusion chromatography, using an Hi-Load Superdex 16/600 column (Cytiva)^25^.

### Circular dichroism analyses

CD spectra were acquired with a Jasco J-1500 spectropolarimeter equipped with a Peltier thermostatic cell holder (Jasco Europe, Cremella (LC), Italy) at 20 °C in the far-UV region from 190 to 260 nm. The peptides **2, 5**, and **6** were analysed at a concentration of 140 µM and the measurements were performed at 20 nm min^−1^scan speed, 1.0 nm band width, D.I.T. 4 s., 0.1 nm data pitch, using a 0.1 cm path length cell in PBS pH 7.4. Each spectrum was obtained by performing three scans, subtracting contributions from corresponding blanks, and converting the signal to mean residue ellipticity in units of deg cm^2^ dmol^−1^ res^−1^.

### Serum stability assays

The proteolytic susceptibility of peptide **5** was determined in 10% (*v:v*) human serum (Lonza, Basel, Switzerland). Human serum was previously activated by cooling at 4 °C, centrifugation at 13,000 ×g for 5 min and incubation at 37 °C for 10 min to eliminate the lipid fraction. Then, 50 μL of serum was added to a 400 μL of ultrapure water and 50 μL of 10 mg/mL peptide mother solution in PBS 10x, thus obtaining a final peptide concentration of 1 mg/mL. The mixture was incubated at 37 °C and, after 0, 1, 2, 4, 6, 24, 48, 72 h, samples (25 μL) were centrifuged at 13,000 ×g for 5 min and 20 μL of supernatant was added to 80 μL of H_2_O containing 0.1% TFA. Supernatants were finally analysed by the LC/MS AGILENT single quadrupole (Agilent 1260 Infinity II LC System) equipped with a diode array detector combined with an electrospray ion source and a quadrupole mass analyser using an Aeris peptide XB-C18 (100 × 4.6 mm; 3.6 μm; 100 Å) and a linear gradient of H_2_O (0.05% TFA)/CH_3_CN (0.05% TFA) from 5 to 70% of CH_3_CN (0.05% TFA) in 30 min at flow rate of 0.8 ml/min. The experiment was run in duplicate.

### Microscale thermophoresis (MST)

MST experiments were performed using a Monolith NT 115 system (Nano Temper Technologies) with LED 100% and IR-laser 20% power. The UBE2C was labelled with NT-647 reactive dyes (Nano Temper Technologies) by N-hydroxysuccinimide (NHS) ester chemistry, which results in efficient reaction with the primary amines of the proteins to form stable dye-protein conjugates, or with red maleimide kit, where maleimide group reacts with cysteine residue s to form a covalent bond^32^. The protein concentration was adjusted to 10 µM with labelling buffer (Nano Temper Technologies), and the labelling reaction was performed by adding the dye in a molar ratio of 1:3 (30 µM). The labelling reaction was incubated for 30 min at room temperature in the dark. The peptides, **2–10** were analysed in the range of the concentration 250 − 0,0038 µM by a 16-step serial dilution 1:2. Measurements were carried out at 25 °C, in 20 mM Tris/HCl, 150 mM NaCl, 0,05% Tween pH 7,5 buffer using premium capillaries. The fit of the normalised fluorescence values versus the ligand concentration was performed by using the software MO-S002 MO Affinity Analysis provided by the manufacturer.

### Interaction studies by alphascreen

AlphaScreen GST Detection Kit was purchased from PerkinElmer and all experiments and incubation were performed in a 384-well optiplate, employing the vendor’s provided assay buffer (PerkinElmer, Waltham, MA). The final concentration of both acceptor and donor beads was 20 μg/ml. For cross-titration experiments, GST- UBE2C and Biotin- **5** were assayed from 10 to 300 nM and they were pre-incubated for 30 min before donor and acceptor beads addition. For competition assay, untagged **5** or **6** peptides were added at 1.65, 3.25, 6.50, 12.50, 25.50 µM. Alpha signal was recorded on Envision 2105 (Perkin Elmer).

### Ubiquitination assay

To assess the activity of recombinant UBE2C, the Ubiquitylation Assay Kit (Abcam, Ab139467) was used. The kit was employed following the manufacturer’s instructions, using UBE2C or UBE2C GST as a substitute for the E2 enzyme. The reaction was performed in a buffer containing 50 mM Tris-HCl (pH 7.5), 2 mM ATP, 5 mM MgCl2, 2 mM DTT, and 0.1 mM EDTA. Additionally, the assay was carried out in the presence of increasing concentration of **5** or **6** peptides, to functionally evaluate their ability to inhibit the ubiquitylation machinery assemblymen. The reaction mixtures were incubated at 37 °C for 3 h, and the UBE2C-Ub complex formation was analysed using SDS-PAGE followed by Western blotting with anti-ubiquitin antibody. Proteins were visualised with an enhanced chemiluminescence detection system (Lite A blot turbo, Euroclone, Milan, Italy) according to the manufacturer instructions and images were acquired with ChemiDoc XRS System (Bio-Rad Laboratories, Italy) and analysed with the QuantityONE software.

### Molecular dynamics (MD) and parallel tempering in well-tempered ensemble (PT-WTE) simulations

The 3D extended (unfolded) structure of peptides **U1**, **2**, **5**, and **6** was first built using the Graphical User Interface (GUI) of Maestro (Schrödinger Release 2025–1) and then parametrised with the ff14SB Amber force field^33^ using the GROMACS 2018.8 code. Each prepared system was then solvated in a 12.0 Å layer cubic box using the TIP3P water model^34^, and Na^+^ was added to neutralise the system’s net charge. Long-range electrostatic forces were calculated with the Particle Mesh Ewald (PME) method, while Van der Waals and short-range electrostatics were used with a cut-off distance of 12 Å. Covalent bonds involving hydrogen atoms were constrained using the LINear Constraint Solver (LINCS) algorithm. Prior to MD, each peptide was first subjected to three minimisation steps with the Polak-Ribiere Conjugate Gradients algorithm and then equilibrated for 20 ns, switching from NPT to NVT ensembles every 1 ns by gradually heating from 50 K to 300 K at 1 bar using the V-rescale thermostat and the Berendsen barostat, respectively. Finally, 20 ns MD production run was performed for each system in NPT ensemble using the Parrinello-Rahaman barostat^35^ with a time step of 2 fs.

The GROMACS 2018.8 code patched with the PLUMED plugin ver. 2.5.3^36^ was used to perform parallel-tempering (PT) combined with well-tempered ensemble (WTE) simulations. PT-WTE is a powerful hybrid technique that enhances the sampling of complex free-energy landscapes, making it particularly suitable for studying small peptides^37–39^, as they exhibit diverse conformations and can switch from one secondary structure to another, such as α-helices, β-sheets, and random coils that are often challenging to sample using conventional MD. In PT, multiple replicas of a system are simulated at different temperatures with configuration exchanges attempted periodically between adjacent replicas based on a Metropolis-like acceptance criterion. Exchange between replicas helps the system escape local minima and improves sampling efficiency. Integrating PT with WTE, facilitates a more efficient exploration of the conformational space by incorporating the system’s potential energy as collective variable (CV). In this way, energy fluctuations across all temperatures are enhanced, allowing a better sampling of the free energy landscape. Furthermore, the WTE bias accelerates transitions between states, allowing for faster convergence of the free-energy calculations. In our case, PT was set up by employing sixteen replicas distributed in temperature range from 300 Kto 500 K, according to the distribution proposed by Prakash et al.^40^^,^^41^ Firstly, each replica was equilibrated with 10 ns of NVT ensemble, without any exchange^42^ Subsequently, the PT-WTE production run was carried out by constructing the metadynamics bias potential on the potential energy CV (E_pot_) so that the Gaussian potential was deposited every 0.5 ps, with a Gaussian width of 312 kJ/mol for peptide **5**, 320 kJ/mol for peptide **6**, 316 kJ/mol for peptide **2** and 320 kJ/mol for the lead peptide **U1**, while the Gaussians initial height was set up to 3 kJ/mol and gradually decreased based on a biasfactor γ = 30^42^. Replica exchanges were set up every 0.5 ps, obtaining an average acceptance probability of 31% for **U1**, 30% for peptide **2,** 30% for **5**, and 29% for **6** between all the neighbour replicas. All replicas were simulated in the NVT ensemble for 250 ns. Only the lowest temperature replica at 300 K was considered for data analysis.

A set of two CVs was selected to reweight the final Free Energy Surface (FES) using the Tiwary–Parrinello algorithm^43^. The choice of a good CV is crucial for accurately describing the folding landscape of small peptides or proteins. Ideally, such CVs should not only allow a clear separation between the relevant end states - namely, the folded (F) and unfolded (U) conformations - but they should also be able to detect metastable intermediates (M) that may emerge along the folding pathway. In fact, selecting suboptimal CVs could lead to the hysteresis phenomenon that can obscure key folding events or misrepresent the free energy surface. In our case, we selected as first CV the dihedral correlation (Dih. Corr.) between all the phi/psi torsion angles of the peptide backbone. It is given by the formula:

$$\text{Dih}.\;\text{corr}.=\frac{1}{2}\;\sum \left[1+\text{cos}\left(\varphi_{i}-\psi_{\text{i }}\right)\right]$$

where the Φ_i_ and ψ_i_ are the torsion angles (Ramachandran angles) of each residue in the peptide chain, so that cos (Φ_i_ - ψ_i_) measures how similar these two angles are. This CV, in fact, measures how consistent or similar are the torsion angles φ and ψ along the sequence and it is particularly effective in distinguishing ordered secondary structures from disordered or unfolded conformations and in detecting metastable states along the folding pathway. The second CV chosen was the AntiBetaRMSD (aβ-RMSD), which can be described by the following formula:

$$a\beta -\text{RMSD}=\sqrt{\;\frac{1}{N}\;\sum_{i=1}^{N}(r_{i}-r_{i}^{\beta }{)}^{2}\;}$$

where *N* is the number of Cα atoms, $r_{i}$ is the position of *i* atom, and $r_{i}^{\beta }$ is the corresponding position of *i* atom in the reference β structure. This CV was designed, in fact, to detect and quantify the formation of antiparallel β-sheet structures of the protein backbone conformation, thus enabling to discriminate between disordered, α-helical and β-strand regions along the folding pathway.

The clustering of the peptides’ conformations in each free energy minima was performed with the GROMOS algorithm of the gmx cluster tool, by considering the RMSD of the peptide backbone using a cut-off of 2.0 Å.

Secondary structure assignments and analysis on the most representative peptide conformation found in each basin were performed with the Dictionary of Secondary Structure in Proteins (DSSP) algorithm^44^ and in-house Python scripts.

Finally, the validation of each peptide conformation was performed by inspecting the psi/phi Ramachandran plot obtained from Maestro GUI (Schrödinger Release 2025–1) (Supplementary Material, Figure S2).

In order to assess the exhaustiveness of our PT-WTE sampling protocol, the convergence of the simulations was evaluated by monitoring the time evolution of the FES at regular intervals of 50 ns, as function of the unbiased Dih.Corr and aβ-RMSD CVs (Supplementary Material, Figures S3-S6). As can be observed from the Supplementary Material, in the last 50/100 ns, the overall shape of the energy landscape remained stable and did not significantly change, thus indicating good qualitative convergence. Secondly, we also evaluated the convergence of the PT protocol by monitoring the good diffusion of all the replicas over all the temperatures (Supplementary Material, Figures S7-S10).

To obtain a more accurate representation of peptide **5** binding to UBE2C in solution, all-atoms molecular dynamics (MD) simulation was performed using GROMACS 2018.8 with the AMBER ff14SB force field for the protein and SPC/E as the explicit water model.^33^^,^^45^. The starting structure corresponded to the best-scoring HADDOCK 2.4 water-refined pose^46^, previously identified as the most representative docking solution for peptide **5**. The complex was placed in an octahedral box with a minimum solute–edge distance of 1.2 nm, solvated with SPC/E water, and neutralised by adding Na^+^/Cl^-^ counterions. For the UBE2C–peptide **5** complex, the geometry was minimised in three consecutive steps: (i) a first steepest-descent minimisation of water molecules and counterions with positional restraints on the solute (up to 50,000 steps), (ii) a second steepest-descent minimisation allowing relaxation of water and solute (up to 100,000 steps), and (iii) a final conjugate-gradient minimisation of the entire system (up to 100,000 steps), using particle mesh Ewald (PME) electrostatics and 1.0 nm short-range cut-offs^47^.

The relaxed system was then equilibrated by means of a multi-step restrained MD protocol totalling 74 ns: an initial NVT stage at 50 K with position restraints on the protein–peptide complex was followed by an NPT stage at 50 K and 1 bar, after which additional NVT/NPT stages of comparable duration were performed, progressively releasing the positional restraints and increasing the temperature to 300 K while stabilising the system density at 1 bar; finally, further NPT simulations at 300 K and 1 bar were carried out to refine density equilibration before starting the production runs.

A 200 ns MD simulation-long was then carried out with a 2 fs time step using a Verlet cut-off scheme and PME for long-range electrostatics, with 1.2 nm short-range cut-offs for both electrostatic and van der Waals interactions^47^. Temperature was maintained at 300 K with a Nosé–Hoover thermostat, and pressure at 1 bar with a semi-isotropic Parrinello–Rahman barostat^35^^,^^48^^,^^49^. All covalent bonds were constrained with the LINCS algorithm^50^. The trajectory was analysed in terms of clustering using the GROMACS gmx cluster tool with the gromos method^51^, employing a 0.6 nm cut-off. Finally, Ubec2 and peptide backbones Root Mean Square Deviation (RMSD) were calculated with GROMACS gmx rmsd tool^52^^,^^53^, and the stability and energetics of the UBE2C–peptide **5** complex were further assessed by estimating binding free energies and per-residue contributions through MM-PBSA calculations using Ambertools23^54^^,^^55^.

### Peptide–protein docking calculations

The binding mode of each peptide against UBE2C was investigated using a computational approach that integrates peptide-protein docking followed by water refinement using the HADDOCK 2.4 web server^46^^,^^56^. First, residues known to be involved in protein-protein recognition were designated as active and used to define ambiguous interaction restraints (AIRs) to guide the docking process. Active residues for UBE2C in HADDOCK calculations comprised those involved in the interactions between the hUbA1 UFD domain and the UBE2C helix H1: P^30^, K^33^, Q^36^, and Q^37^ in UbcH10.

HADDOCK calculations yielded 10 clusters for each peptide; the top-scoring conformations were subsequently refined using the HADDOCK 2.4 water refinement protocol to enhance structural accuracy. The binding free energies (ΔG) of the top-scoring refined peptide–protein complexes were estimated using the PRODIGY web server^57^. This server predicts ΔG (kcal·mol^−1^) and K_d_ (at 25 °C) based primarily on the number and nature of intermolecular contacts within 5.5 Å, as well as the characteristics of the non-interacting surface.

## Results

### Folding of U1 peptide in aqueous solution

For the rational design of a new generation of **U1** derivatives, we first asked what folding conformation of the parent peptide **U1** was predominant in water solution instead of the X-ray environment. Thus, to accurately sample the conformational states of the lead peptide **U1**, we employed the PT-WTE approach as described in the Materials and Methods section. Integrating PT with WTE facilitates a more efficient exploration of the conformational space by incorporating the system’s potential energy as a CV. The choice of a good CV is crucial for accurately describing the folding landscape of small peptides or proteins, including not only the folded and unfolded conformations, but also metastable intermediates that may emerge along the folding pathway. In our protocol, the FES of **U1** was reweighted as a function of Dihedral Correlation (Dih. Corr.) and aβ-RMSD CVs (see Materials and Methods section). The resulting reweighted FES of **U1** (Supplementary Material, Figure S11) revealed a single energetically favoured minimum A of ∼ −3.20 kJ/mol, corresponding to an unfolded *(U)* coil/turn state with only two residues D^1042^ and E^1043^ adopting partial 3_10_-helices character (∼40%). This unfolded state *(U)* was then followed by a ∼7 kJ/mol energetically higher misfolded *(M)* metastable basin B with a prevalence of coil/turn/*β*-bridge motifs. In contrast, conformations adopting a folded *(F)* antiparallel β-sheet secondary structure were located in basin C (nearly isoenergetic with basin B), representing the highest free energy regions of the reweighted FES. Here, the β-sheet state was formed at residues C^1039^-N^1041^ and D^1047^-E^1049^, mediated by two hydrogen bonds with the backbone of C^1039^-V^1048^ and C^1040^-V^1048^ that stabilise the β-sheet motif along the hydrophobic core of L^1038^, C^1039^, C^1040^, V^1048^, and V^1050^ (Supplementary Material, Figure S11). Remarkably, structural superposition between the X-ray conformation of **U1** (U1*_xr_*) and the most representative cluster within basin C of the PT-WTE simulation revealed an excellent spatial overlap with the backbone atoms (RMSD = 0.78 Å) (Supplementary Material, Figure S12). This indicates that the β-sheet conformation observed in the crystal structure (U1*_xr_*) corresponds to a well-defined, but high-energy, conformational state in aqueous solution, likely stabilised in the crystal but less populated under physiological conditions.

On the basis of these findings, we designed a new generation of **U1** derivatives, peptides **2**–**10**, to improve the physical-chemical properties through strategic residue substitutions, focusing on four key aspects: *(i)* the conformational behaviour, by preserving the predominant and energetically-stable aqueous-phase secondary structure folding (α-helix/β-sheet/coil) of **U1** in the modified sequences; *(ii)* improving the stability reducing spontaneous aggregation of **U1** through strategic residue substitutions; *(iii)* Introducing chromophores to obtain a more precise quantification through UV absorbance; *(iv)* identify the hot-spot charged amino acids likely essential for the interaction with the H1 helix interface of *h*UbcH10.

### Optimising the physical-chemical properties of U1-derived peptides

Firstly, to overcome the problem of the observed spontaneous aggregation of the **U1** peptide in solution, the first derivative of **U1**, the peptide **2**, was designed by replacing the two C^1039^ and C^1040^ residues with two serine, S^1039^ and S^1040^, in order to increase the hydrophilicity and solubility of the peptide in aqueous environments. Another aspect considered in the design of the new derivatives was the evaluation of the impact of the incorporation of amino acids with aromatic side chains, as terminal modifications such as N-terminal or C-terminal aromatic tags as substitutions in the sequence^58^. With this in mind, a series of derivatives of peptide **2** were designed incorporating tryptophan tags at the N-term (W^1037^) or at the C-term (W^1051^), yielding peptides **3** and **4**, respectively. Moreover, the L1038W and E1046Y substitutions have been introduced, leading to peptides **5** and **6**, respectively. Finally, the final aspect considered in our design strategy was the prediction of hot-spot residues interacting with the H1 helix of hUBCh10.

**Figure 2.**
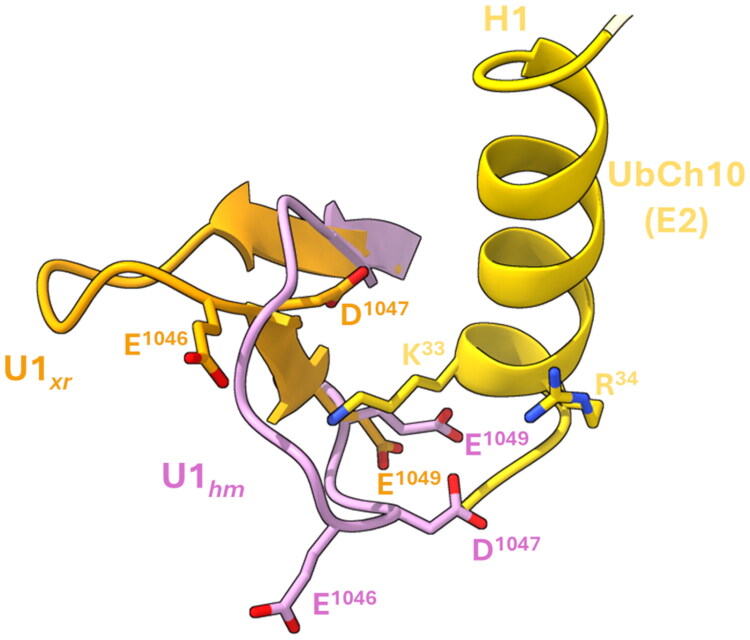
Overlap between the X-Ray β-sheet conformation of U1*_xr_* (PDB ID: 6DC6) (orange cartoon) with that of the homology model β-turn of U1*_hm_* (plum cartoon)^31^ with their interaction with the H1 helix of hUbcH10 (yellow cartoon). Negatively charged amino acids E^1046^, D^1047^ and E^1049^, reported to be involved in E1–E2 interactions^59^ within the H1 residues, are represented in sticks.

The analysis of the U1*_hm_* conformation in the computational homology model of the *h*UBA1/*h*UbcH10 complex^31^ suggested, as putative hot-spots, the negatively charged residues E^1046^ and E^1049^ since they are close enough to engage electrostatic interactions with K^33^ and R^34^ of the H1 helix of *h*UbcH10 (Figure 2). To experimentally validate this prediction, we assessed whether substituting these glutamate residues with polar, uncharged amino acids would disrupt the activity against hUbcH10. Accordingly, we generated the variants E1046Y, E1046Q, and E1049N, yielding peptides **6**, **7**, and **8**, respectively. In the final stage of our design, we integrated elements from phases (ii), (iii), and (iv) by introducing W^1037^ as an N-terminal tagging residue to peptide **2,** and we have incorporated mutations D1042N, E1043Q, and D1047N leading to peptides **9**, **10** and **11** (Table 1), to investigate the role of these negatively charged residues in mediating the interaction with the H1 helix of hUbcH10. All peptides were obtained, as described in the Materials and Methods section, in good yields (ca. 40%), and their identity and purity were assessed by SQ LC/MSD (Table 1).

### Protein expression and characterisation

Recombinant UBE2C was expressed and purified as previously described^31^, using a pGEX-4T1 expression vector that generates an N-terminal GST fusion protein. Protein production was carried out in *E. coli* BL21 (DE3) RP cells induced with IPTG at low temperature to promote proper folding. Following cell lysis and clarification, the fusion protein was recovered by affinity chromatography using a GST-trap column. The GST tag was then removed by proteolytic cleavage with thrombin, and UBE2C was further purified by a second GST-trap step to eliminate residual tag and by size-exclusion chromatography to obtain the final monomeric fraction. The overall yield of purified UBE2C was approximately 2 mg/L, with a purity exceeding 95% as assessed by SDS-PAGE.

To ensure that the recombinant protein retained enzymatic activity, an *in vitro* ubiquitylation assay was performed (Supplementary Material, Figure S13). This test confirmed the catalytic competence of UBE2C and, importantly, demonstrated that the presence of the GST tag does not impair its ability to form ubiquitin-conjugated complexes under standard reaction conditions. These results validate the use of either protein form in subsequent binding or enzymatic assays^25^.

### Binding properties of U1-derived peptides evaluated by MST

**Figure 3.**
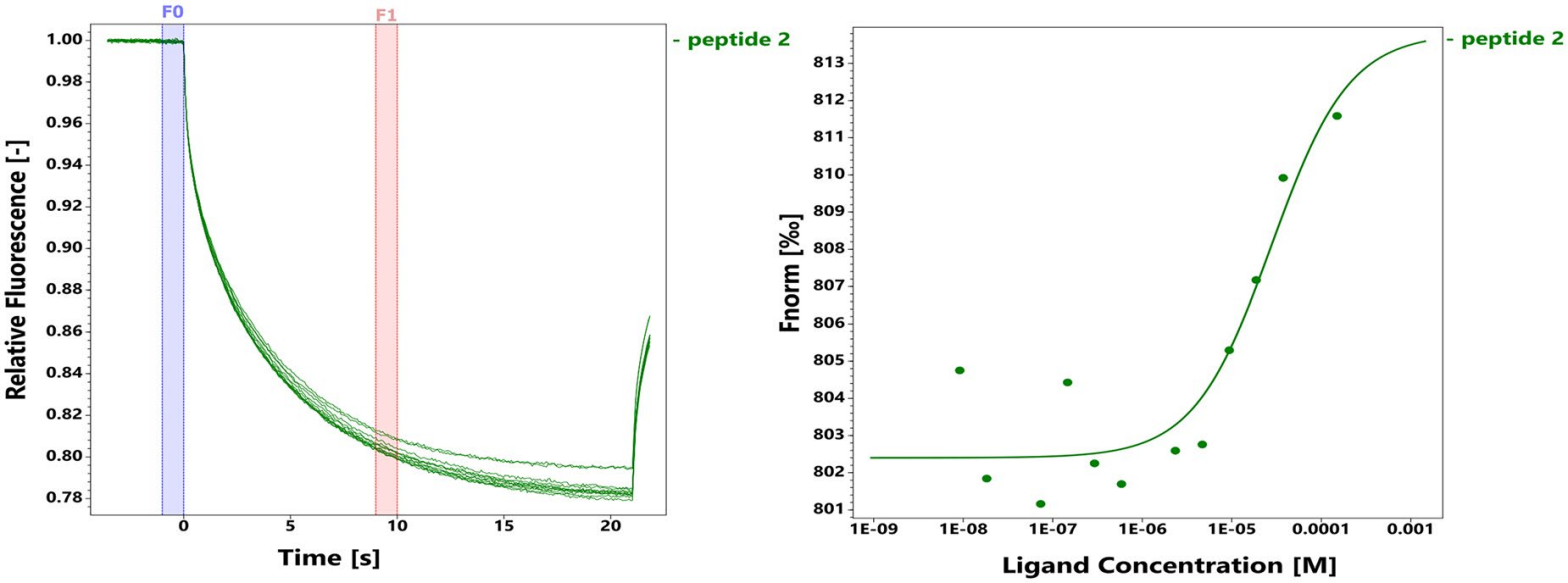
MST interaction analysis of **2** against UBE2C. MST traces (left) and dose-response curves (right) of **2** titrated against UBE2C.

To validate our peptide design strategy, we first investigated whether the substitution of cysteine residues at positions C1039 and C1040 with serine (C1039S, C1040S), present in peptide **2**, preserved its capability to bind UBE2C. This substitution was selected to verify its potential as a viable starting point for developing new peptide inhibitors of UBE2C. Thus, peptide **2** was synthesised and its binding properties were evaluated through MST experiments. The MST assays showed that peptide **2** binds to UBE2C with micromolar affinity, exhibiting a dissociation constant (K*_d_*) of approximately 27.7 μM (Figure 3). These results confirmed that the replacement of cysteine residues with serine only partially affected the peptide’s ability to interact with UBE2C, suggesting that the structural integrity and essential binding determinants of the peptide remained largely intact despite these substitutions.

Encouraged by this finding, we proceeded to design and test a series of derivatives based on peptide **2** (Table 1), aiming to systematically identify residues critical for binding and potentially enhance the affinity through selective amino acid modifications. The binding properties of these newly synthesised derivatives were again assessed by additional MST assays, and the results (summarised in Table 2 and detailed in Figure 3 and in Figure S14 of the Supplementary Material) provided crucial insights into the structure–activity relationships governing the interaction between these peptides and UBE2C. Among these variants, the introduction of a tryptophan residue, a modification implemented both to improve peptide visualisation and quantification, yielded enhanced interaction only when L^1038^ was replaced by tryptophan (L1038W, peptide **5**). Remarkably, peptide **5** displayed a binding affinity (K*_d_* = 10 μM) comparable to the reference peptide **U1**, clearly highlighting the positional sensitivity of this residue substitution. Interestingly, peptide **10**, in which tryptophan was added at the N-terminus (W^1037^) and glutamine replaced glutamate at position 1043 (E1043Q), retained a measurable, though reduced, binding affinity (K*_d_* = 40 μM). This result indicated that the presence of an N-terminal tryptophan does not impair binding, and that residue E1043 was not strictly essential for UBE2C interaction. Conversely, the addition of tryptophan at the C-terminus (W^1051^), as in peptide **4**, did not result in any detectable interaction, indicating that positional context plays a crucial role in the functional effect of this residue.

**Table 2.** Aminoacidic sequences of **U1** and of the newly designed peptides with K*_d_* values determined by MST experiments.

Peptide	Sequence and residues number															K*_d_ (*µM)
	1037	1038	1039	1040	1041	1042	1043	1044	1045	1046	1047	1048	1049	1050	1051	
**U1(1)**		L	C	C	N	D	E	S	G	E	D	V	E	V		^a^
**2**		L	**S**	**S**	N	D	E	S	G	E	D	V	E	V		**27.7**
**3**	**W**	L	**S**	**S**	N	D	E	S	G	E	D	V	E	V		***n.d.***
**4**		L	**S**	**S**	N	D	E	S	G	E	D	V	E	V	**W**	***n.d.***
**5**		**W**	**S**	**S**	N	D	E	S	G	E	D	V	E	V		**10**
**6**		L	**S**	**S**	N	D	E	S	G	**Y**	D	V	E	V		***n.d.***
**7**		L	**S**	**S**	N	D	E	S	G	**Q**	D	V	E	V		***n.d.***
**8**		L	**S**	**S**	N	D	E	S	G	E	D	V	**N**	V		**220**
**9**	**W**	L	**S**	**S**	N	**N**	E	S	G	E	D	V	E	V		***n.d.***
**10**	**W**	L	**S**	**S**	N	D	**Q**	S	G	E	D	V	E	V		**40**
**11**	**W**	L	**S**	**S**	N	D	E	S	G	E	**N**	V	E	V		***n.d.***

^a^ 10 µM from Elisa assay in our previous paper [25]; n.d.= not determined

In contrast, substitutions involving acidic residues D^1042^, E^1046^, and D^1047^ proved detrimental to the interaction with UBE2C. Indeed, peptides such as **6**, **7**, and **11**, in which these acidic residues were altered, exhibited a complete loss of binding capability, thereby underscoring the essential structural and electrostatic roles played by these residues in stabilising the interaction interface. Based on these MST findings, peptide **5** emerged as the most promising candidate for subsequent studies, given its optimised binding affinity coupled with minimal structural alterations. Conversely, peptide **6**, characterised by the substitution E1046Y, showing no detectable binding, was selected as a suitable negative control for further binding specificity assays.

### Validation of peptide–UBE2C interactions by AlphaScreen assays

**Figure 4.**
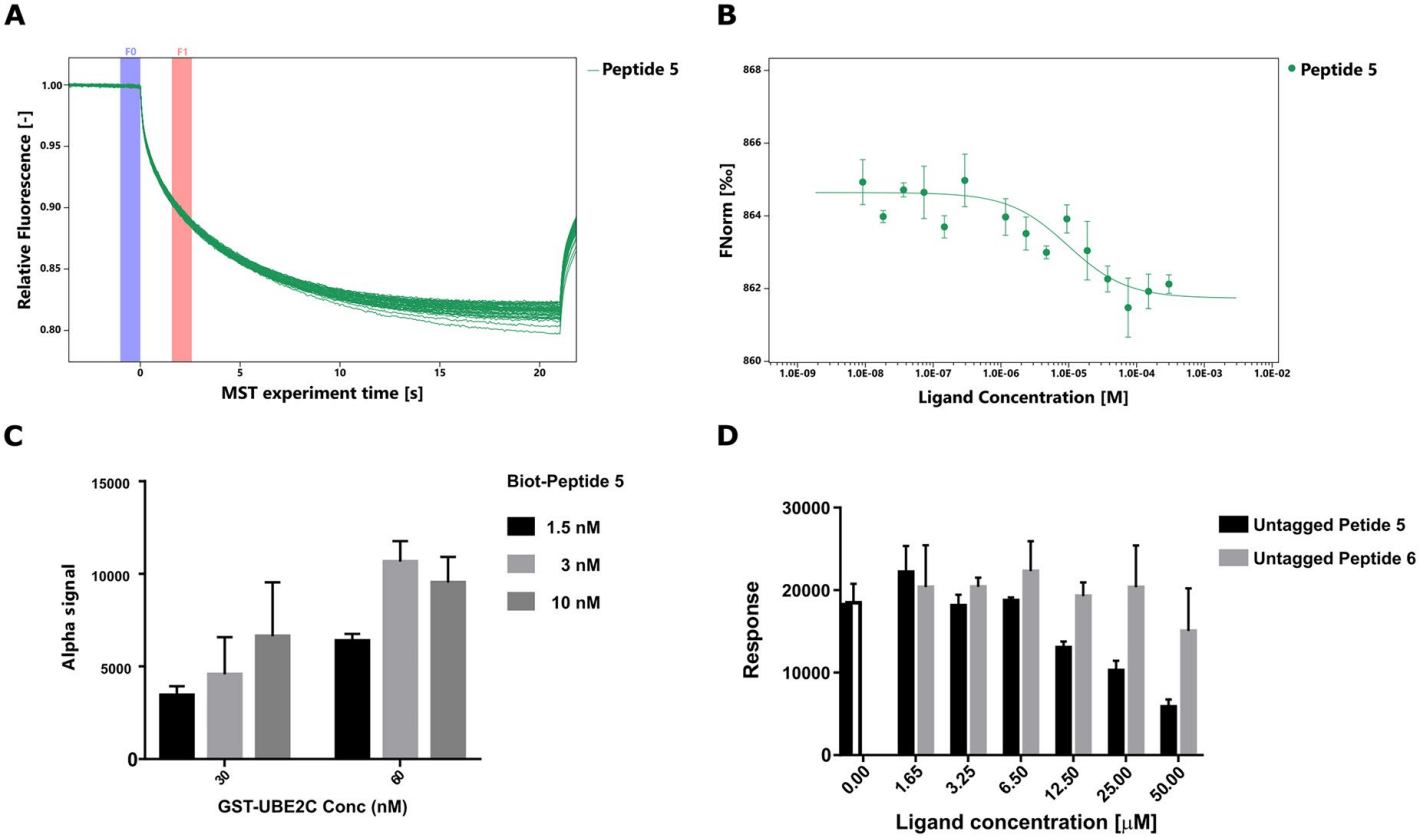
Binding Peptide 5 assays: (A) MST traces of UBE2C in the presence of different concentrations of **5** peptide. (B) Binding curve obtained by plotting the fluorescence normalised signal versus increasing concentration of **5**. (C) AlphaScreen- Cross-titrations experiments. (D) AlphaScreen competition experiments using **5** and **6** peptides.

To further validate the binding properties of the most promising peptide identified by MST (Figure 4(A,B), and Supplementary Material Figure S14), the ability of peptide 5 to bind UBE2C was assessed also with AlphaScreen assays in a competitive binding configuration. Given the different physical principles underlying AlphaScreen and MST, the use of this additional assay provided an independent evaluation of the binding behaviour. Peptide **6**, which had shown no measurable binding in MST, was included as a negative control to confirm the specificity of the interaction.

AlphaScreen assays are highly sensitive to biomolecular interactions and can detect a broad range of affinities, with dissociation constants spanning from pM to low µM. However, direct binding assays may lead to an overestimation of the apparent affinity due to the avidity effect of the beads and the presence of multiple binding sites. To correct this effect, we performed competition experiments employing one of the interactors in the untagged form, allowing for an accurate estimation of the binding affinity.^60^

In our experimental setup, UBE2C was expressed as a GST-tagged fusion protein, while peptides were synthesised with a biotin modification. Cross-titrations of GST–UBE2C and biotinylated **5** were first performed to define the optimal conditions for signal detection and avoid the so-called “Hook effect”, a decrease in signal due to beads saturation. As shown in Figure 4(C), the highest signal was obtained with 60 nM UBE2C and 3 nM biotinylated **5** peptide, and these concentrations were used in subsequent competition assays. Competition experiments were then performed by incubating increasing concentrations of the untagged peptides **5** or **6** in the presence of GST-UBE2C and biotin-**5**. As shown in Figure 4(D), untagged-**5** was able to bind to GST-UbcH10, competing with beads-immobilised Biotin-**5** and causing a concentration-dependent reduction in Alpha signal. In contrast, the control peptide **6** did not show the same behaviour, indicating that the interaction of **5** was sequence specific. These results therefore confirmed the MST data and provide consistent support for the binding competence of **5**, reinforcing its identification as promising lead candidate for UBE2C targeting.

### Conformational behaviour of peptides 2, 5 and 6

**Figure 5.**
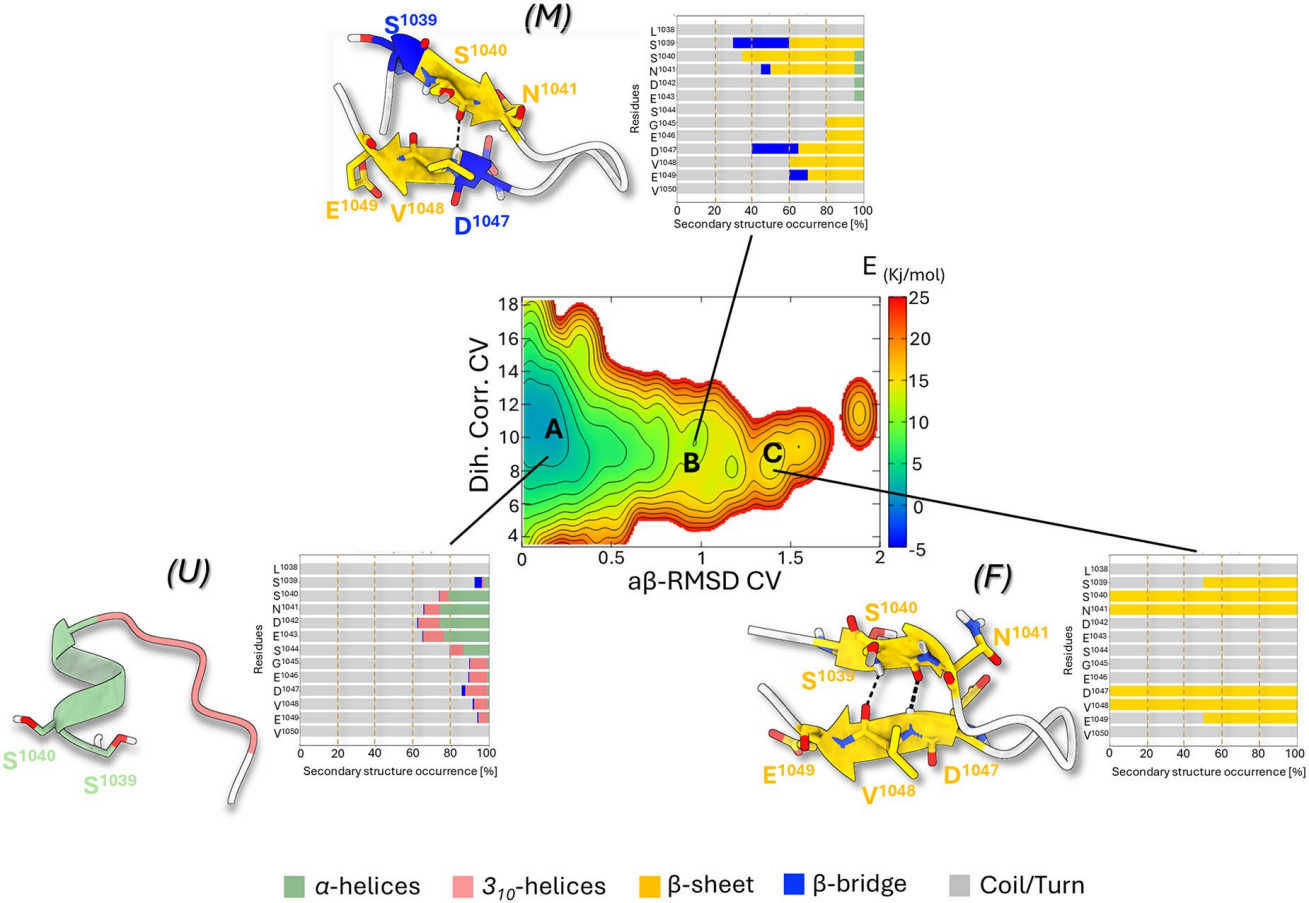
Free energy surface (FES) reweighted as a function of Dihedral Correlation (Dih. Corr.) and AntiBeta RMSD (aβ-RMSD) CVs representing the folding process of peptide **2**. Isosurfaces are displayed every 5 kJ/mol. The representative conformation extracted from the main free energy minima is shown on the left and is coloured for the secondary structure folding: α-helices and *3_10_*-helices regions are coloured as light-green and pink cartoon, respectively, β-sheet and β-bridge states are coloured as yellow and blue cartoon, respectively, while coil/turn unfolded regions are coloured as light-grey cartoon. Adjacent bar plots indicate the occurrence of different secondary structure motifs at residue level throughout the ensemble of basins.

**Figure 6.**
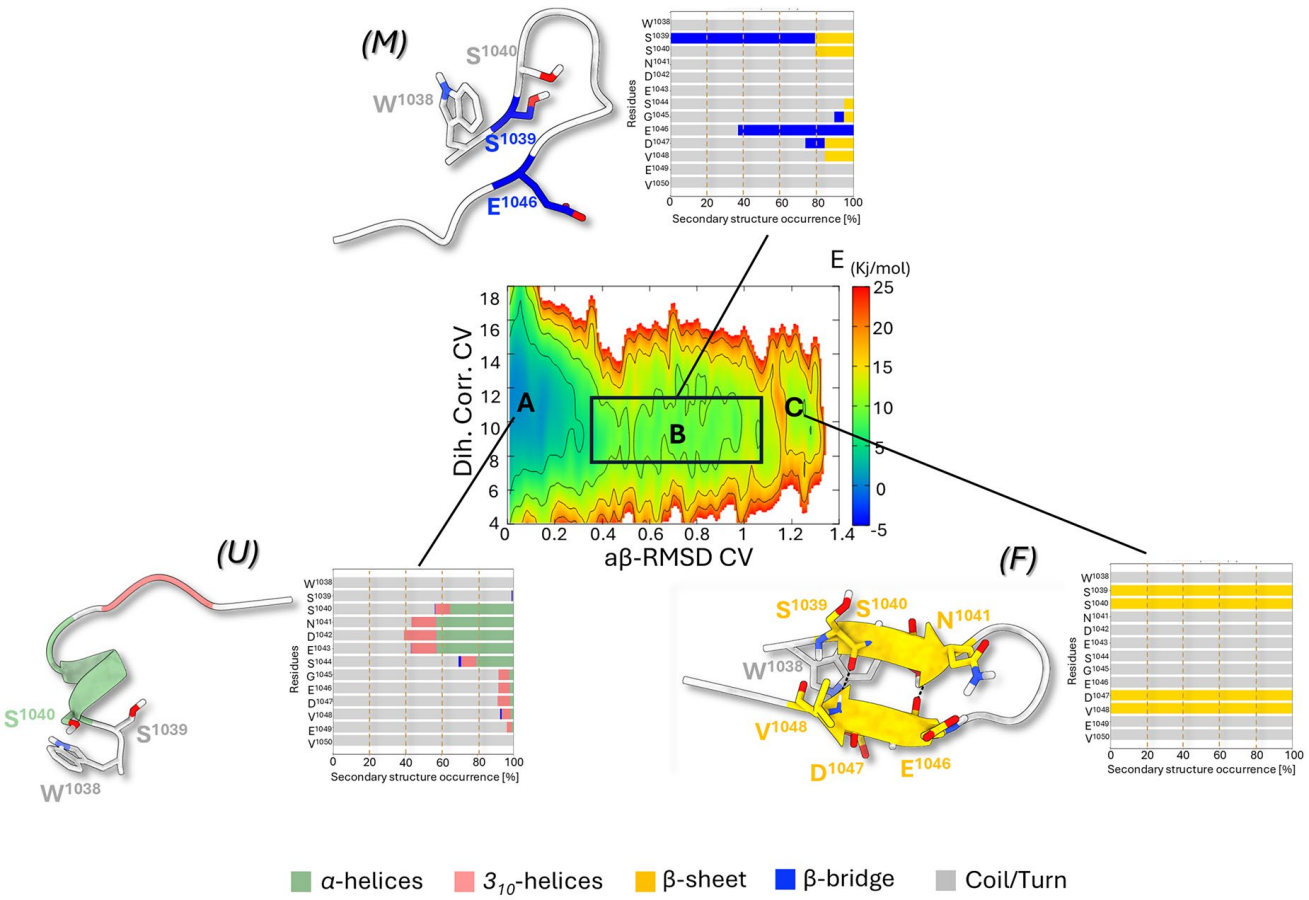
Free energy surface (FES) reweighted as a function of Dihedral Correlation (Dih. Corr.) and AntiBeta RMSD (aβ-RMSD) CVs representing the folding process of peptide **5**. Isosurfaces are displayed every 2 kJ/mol. The representative conformation extracted from the main free energy minima is shown on the left and is coloured for the secondary structure folding: α-helices and *3_10_*-helices regions are coloured as light-green and pink cartoon, respectively, β-sheet and β-bridge states are coloured as yellow and blue cartoon, respectively, while coil/turn unfolded regions are coloured as light-gray cartoon. Adjacent bar plots indicate the occurrence of different secondary structure motifs at residue level throughout the ensemble of basins.

**Figure 7.**
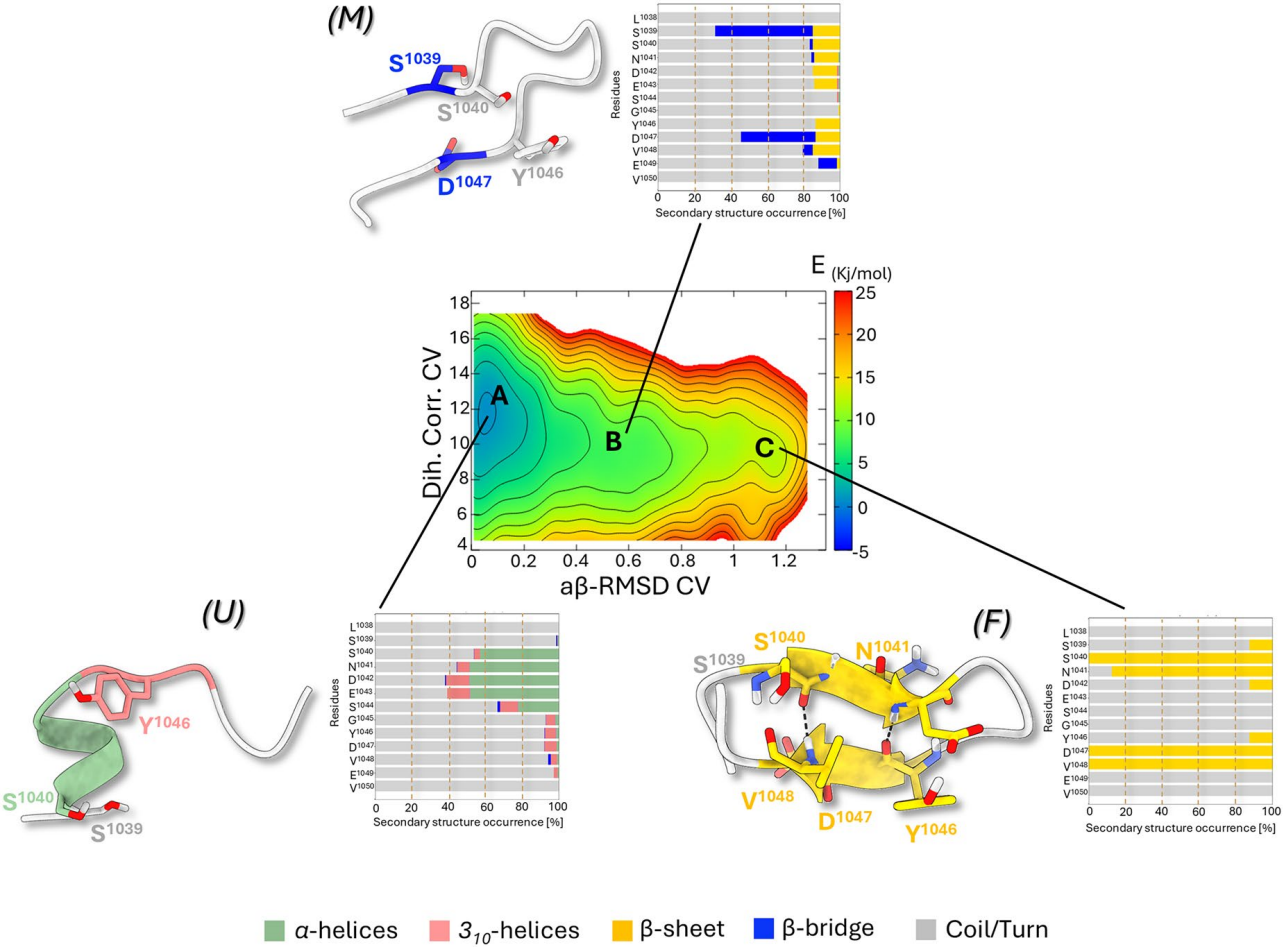
Free energy surface (FES) reweighted as a function of Dihedral Correlation (Dih. Corr.) and AntiBeta RMSD (aβ-RMSD) CVs representing the folding process of peptide **6**. Isosurfaces are displayed every 2 kJ/mol. (A-C) The representative conformation extracted from the main free energy minima is shown on the left and is coloured for the secondary structure folding: α-helices and *3_10_*-helices regions are coloured as light-green and pink cartoon, respectively, while β-sheet and Coil/turn folding regions were coloured as yellow and light-grey cartoon, respectively. Adjacent bar plots indicate the occurrence of different secondary structure motifs at residue level throughout the ensemble of basins.

To explore the influence of the mutations on the folding behaviour of the most promising designed peptides **2** and **5** and the negative control **6**, we performed PT-WTE simulation using the same protocol adopted for the parent peptide **U1** and described in the Materials and Methods section. The reweighting of the 2D-FES reported in Figures 5–7 revealed that in all three peptides it was dominated by one main minimum A located at low aβ-RMSD CV values (0.0–0.3) and high Dih. Corr. CV values (∼11). This region corresponded to a largely unfolded *(U)* ensemble with minimal local stable secondary structure elements and predominant coil/turn motifs limited to only 1-turn α-helical or 3_10_-helix formation, indicating a high degree of structural plasticity. Specifically, 1-turn α-helix formation spanning residues S^1040^-E^1043^ was more frequent in **5** and **6** (∼60%) compared to peptides **2** (∼40%) and **U1** (< ∼40%). These U states preceded the intermediate metastable states (M), which were energetically higher by about 10 kJ/mol than basins B characterised by the formation of more ordered secondary structure organisation. As the aβ-RMSD CV increases, in fact, all three peptides explored conformations with partially folded *β*-bridge motifs closer to a β-sheet-like geometry. This was particularly evident for peptides **2** and **6**, which tend to adopt a *β*-bridge conformation in proximity to S^1039^ and D^1047^ (Figures 5 and 6, respectively). However, compared to the wild-type **U1** peptide, we observed that mutations altered the propensity of the peptides to transition from the U to the F states. This was evident from the 1D-FES comparison between **U1** and **2** (Supplementary Material, Figure S15), where the substitution of the two cysteines with serines resulted in an ∼8 kJ/mol increase in the energy barrier between the M and F states in **2,** thus hampering the propensity of this peptide to overcome the transition state (TS) and reach the folded (F) state.

In contrast, the rugged free energy landscape observed for peptide **5**, characterised by multiple energy barriers and local structural ensemble between the unfolded and the folded states (mid-aβ-RMSD range, Figure 6), indicated the transient sampling of partially folded intermediates without a single well-defined β-sheet basin. However, the mutation L1038W reshaped the free energy landscape, lowering the transition barriers and extending the M state to the F state, thus facilitating the progression along the U→M→F folding pathway. Consequently, **5** an increased ability to overcome energy barriers, enhancing the frequency with which it sampled conformations resembling the *β*-sheet motif, even though these states did not form a distinct stable basin. This was in line with the energetically lower 1D-FES observed. The presence of tryptophan at the peptide’s N-terminus, in fact, could introduce steric hindrance that influenced the local backbone conformation, favouring transient β-sheet formation under the simulated conditions. This peculiar folding behaviour of **5** could indirectly influence its binding to UbCH10. The presence of an extended metastable intermediate (M) along the U → F folding pathway might, in fact, suggest a *fly-casting*–like mechanism of binding^61^, in which the partially unfolded *U* state could promote the early recognition and the binding to UbcH10, facilitating long-range interactions then followed by conformational locking into the native F state.

On the contrary, the 2D-FES profile of the negative control **6** (Figure 7) was characterised by partially folded or unfolded (random coil) conformations in basin A, closely matching those of **6**, with a 1-turn α-helix encompassing residues S^1040^-E^1043^. Thus, the E1046Y mutation does not significantly alter the conformations of basin A but rather the U→F folding pathway. Looking at the 2D-FES, it was evident that only transient misfolded conformations (M) were observed, with not well-defined intermediate basins, suggesting a more continuous U→F process with fewer metastable states. This peculiar conformational behaviour was not liable for loss of binding activity against hUBa1, which, instead, could be due to the loss of the E^1046^ negative charge and its substitutions with bulkier Y^1046^ as demonstrated by further docking calculations.

### Experimental validation of peptide conformational behaviour

To experimentally validate the conformational features emerging from the PT-WTE simulations of peptides **2**, **5**, and **6**, we performed far-UV circular dichroism (CD) spectroscopy under the same solution conditions used in the MST binding assays. This analysis aimed to assess whether the structural propensities observed computationally, namely the predominance of unfolded or partially folded ensembles, were also reflected in the solution-state secondary structure of the peptides. As shown in Figure S16 of the Supplementary Material, all three peptides exhibited CD spectra characteristic of predominantly disordered conformations, with a negative band below 200 nm and no evident minima at 208 or 222 nm, typical of α-helical content.

### Docking calculations

**Figure 8.**
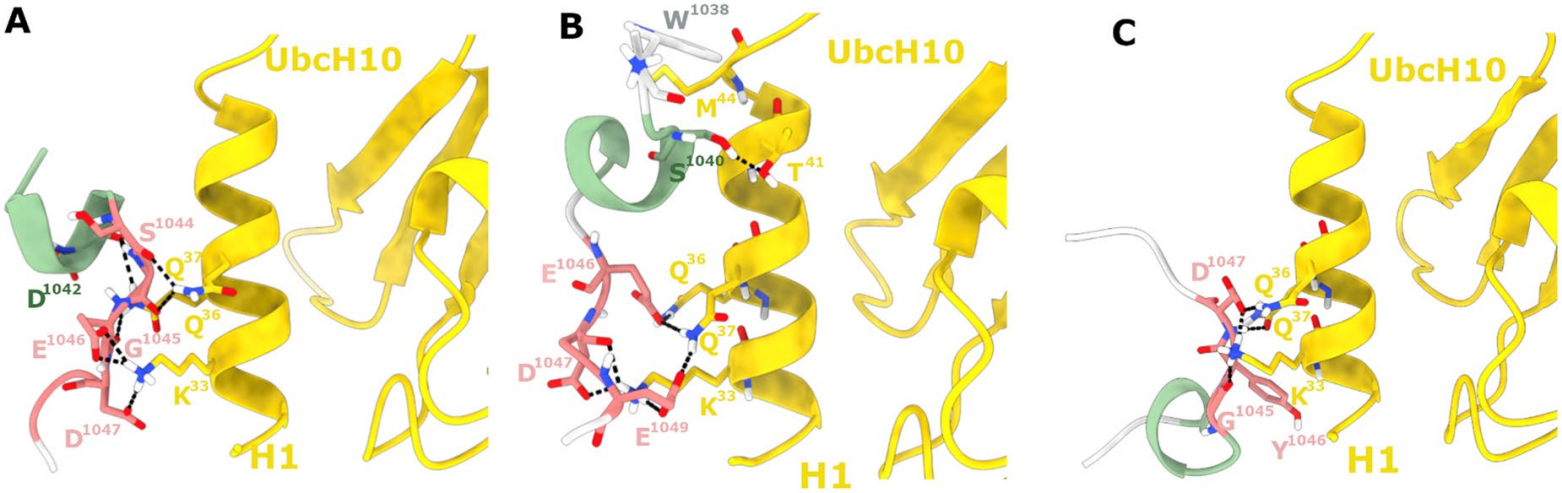
Best docking poses of PT-WTE conformations found in basin A of peptides: (A) **2**, (B) **5**, and (C) **6** against UBE2C as obtained from HADDOCK 2.4. UBE2C is shown as a yellow cartoon, while the peptides are visualised in cartoon format and coloured according to their secondary structure, following the scheme used in the PT-WTE analysis: α-helices and 3_10_-helices in light green and pink, β-sheets and β-bridges in yellow and blue, and coil/turn regions in light grey. Interacting residues are shown as sticks and labelled; hydrogen bonds are represented as black dashed lines.

The binding mode of peptides **2, 5**, and **6** was investigated by peptide–protein docking through a two-step procedure using the HADDOCK 2.4 web server with a final estimate of ΔG of each complex calculated from the PRODIGY web server, in order to have a direct comparison of docking results (Supplementary Material, Table S1 and S2). The partially unfolded lowest energy conformations found in basin A from PT-WTE calculations were used as input structures. In particular, a rigid-body docking was performed, followed by a flexible water refinement step applied to the top-scoring conformation in order to improve the structural accuracy of the binding mode prediction. The best scored docking pose for each peptide is shown in Figure 8.

Specifically, **2** interacts with helix 1 (H1) of UBE2C, where the side chain of K^33^ forms hydrogen bonds with the side chains of E^1046^ and D^1047^, Q^36^ establishes hydrogen bonds with the backbone atoms of D^1042^ and E^1046^, while Q^37^ interacts with the backbone of S^1044^ and G^1045^ (Figure 8(A)). These interactions stabilise the peptide–protein complex, as reflected by a ΔG of −6.4 kcal/mol estimated from the PRODIGY webserver (Supplementary Material, Table S2).

Replacing L^1038^ with W^1038^ as in the **5** (Figure 8(B)) slightly rearranges the binding mode with respect to **2,** improving the overall binding affinity. Notably, K^33^ of UBE2C forms hydrogen bonds with both the backbone and the side chain of D^1047^, as well as with the side chain of E^1049^. Furthermore, Q^36^ forms a hydrogen bond with the side chain of E^1046^ and Q^37^ establishes multiple hydrogen bonds with the side chains of both E^1046^ and E^1049^, which further stabilises the interface. T^41^ also forms a further hydrogen bond with the side chain of S^1040^, providing an additional anchoring point. The W^1038^ introduced at the N-terminus contributes to binding through hydrophobic interactions with M^44^ of UBE2C, enhancing the peptide’s overall affinity for the target, as demonstrated by the improved ΔG obtained for **5** (−7.0 kcal/mol) (Supplementary Material, Table S2), compared to peptide **2**, reflecting a more favourable binding affinity and correlating with experimental data.

The binding interface of peptide **6** (Figure 8(C)) differs significantly from those observed for the **2** and **5** peptides. In this conformation, K^33^ forms hydrogen bonds with the backbone of G^1045^ and the side chain of D^1047^, Q^36^ interacts with the backbone of D^1047^ and Q^37^ establishes a hydrogen bond with the side chain of D^1047^. However, replacing E^1048^ with a tyrosine residue leads to a reduction in stabilising contacts, weakening the overall peptide–protein interaction. This was evident from the less favourable ΔG of −6.2 kcal/mol. This lower binding affinity was consistent with the lack of biological activity observed for **6**, supporting the importance of E^1048^ in mediating stable and functional interactions with UBE2C.

### Molecular dynamics simulations of the peptide 5 binding mode

**Figure 9.**
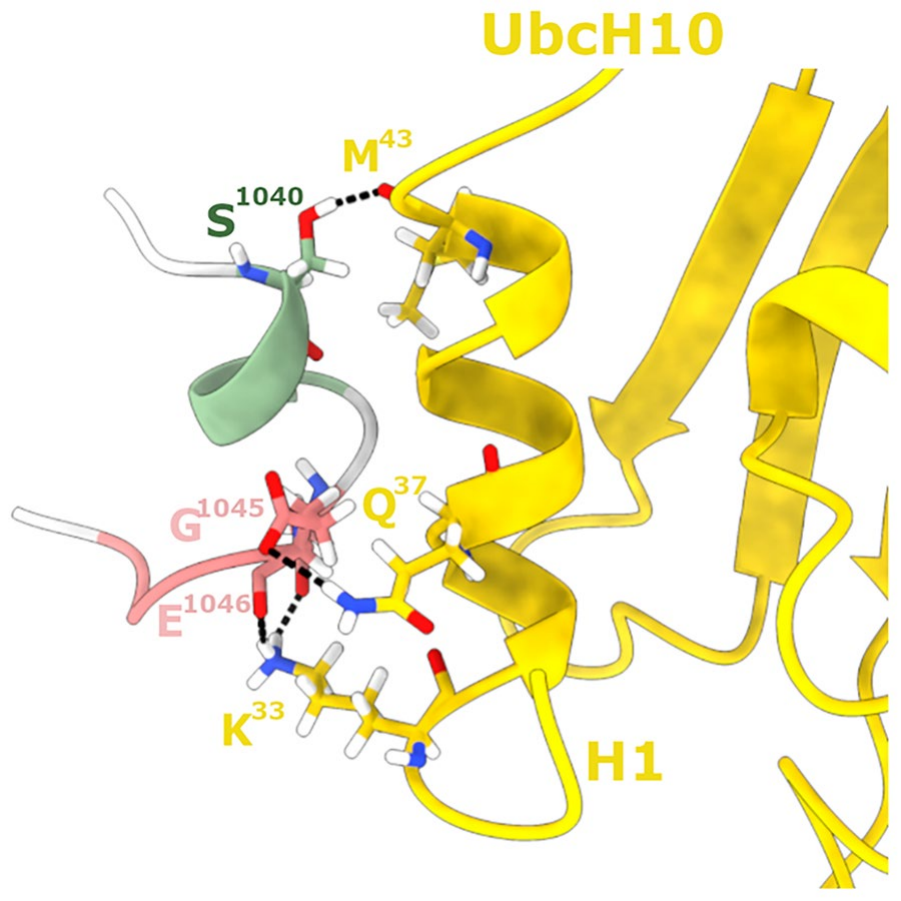
Representative binding mode of peptide **5** on UBE2C obtained from MD simulations. The structure corresponds to the centroid of the most populated cluster (cluster 0) extracted after the200-ns MD trajectory. UBE2C is shown as a yellow cartoon, while the peptide is visualised in cartoon format and coloured according to its secondary structure, following the scheme used in the PT-WTEanalysis: α-helices and 3_10_-helices in light green and pink, β-sheets and β-bridges in yellow and blue, and coil/turn regions in light grey. Interacting residues are shown as sticks and labelled; hydrogen bonds are represented as black dashed lines.

A 200 ns-long molecular dynamics (MD) simulation was performed on peptide **5**, which has emerged as the most active and promising candidate in the experimental assays, in order to validate its binding mode and quantify its affinity towards UBE2C. The Root Mean Square Deviaton (RMSD) profile computed on the UBE2C backbone (Supplementary Material, Figure S18) indicates a highly stable protein throughout the simulation, with an average RMSD of 1.84 Å. In contrast, peptide **5** shows a short equilibration phase during the first ∼15 ns, after which it reaches a stable binding mode that is maintained for the remainder of the trajectory, yielding an average RMSD of 9.30 Å. This value reflects the intrinsic flexibility of the peptide, as the structured α-helical region remains well-defined and stable, whereas the unfolded loop portion exhibits greater mobility. Cluster analysis of the peptide backbone, performed using the gmx cluster tool of GROMACS^51^, identified three predominant clusters with populations of 33%, 16%, and 14% (Figure 9 and Figure S17). Notably, the centroid of the most populated cluster (Figure 9) retains a binding mode highly similar to one of the docking pose, with nearly all key intermolecular interactions preserved throughout the simulation. Specifically, peptide **5** engages the H1 region of UBE2C through a network of stabilising contacts: the side chain of K^33^ forms hydrogen bonds with the backbone atoms of G^1045^ and E^1046^ and establishes a salt bridge with E^1046^; Q^37^ forms a hydrogen bond with the side chain of E^1046^; the backbone of M^43^ engages hydrogen bonds with the side chain of S^1040^. Together, these interactions strongly stabilise the binding interface.

Moreover, the cluster analysis clearly shows that, across all major clusters, the structured α-helical segment preserves nearly identical coordinates and reproduces the same interaction network with UBE2C, whereas the variability arises almost exclusively from the unstructured loop, which is not directly involved in the core binding interface. This strongly supports the presence of a well-defined and persistent binding mode.

The stability of the interaction is further strengthened by MM/PBSA binding free energy calculations. The computed total binding free energy (ΔG_bind_ = −359.31 kJ/mol) strongly supports a robust and thermodynamically favourable complex. The dominant stabilising contribution arises from electrostatic interactions (ΔE_EEL = −1806.67 kJ/mol), which remain highly favourable even after compensation by the polar solvation term. In addition, both van der Waals interactions (ΔE_VDW = −32.48 kJ/mol) and non-polar solvation effects contribute cooperatively to the binding stabilisation. The convergence of these energy components unequivocally indicates that peptide **5** engages UBE2C through a tightly stabilised interface dominated by persistent electrostatic contacts and complemented by hydrophobic packing.

Taken together, the MD trajectory, clustering analysis, and MM/PBSA energetics consistently demonstrate that peptide **5** forms a stable and well-defined complex with UBE2C, fully supporting its superior performance observed experimentally.

### Ubiquitylation assay

**Figure 10.**
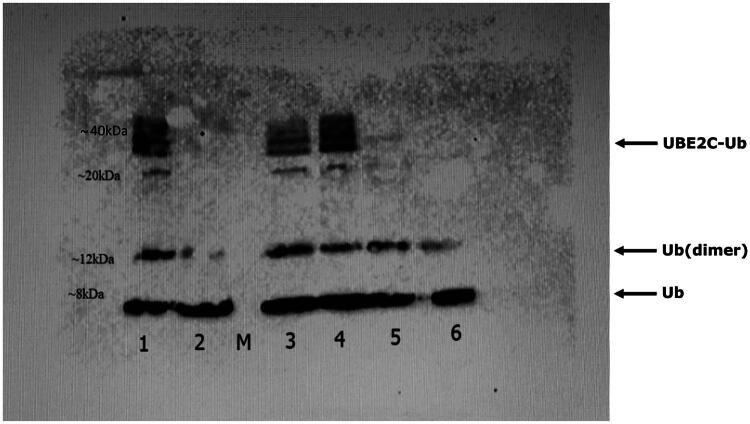
Western blot analysis of E2-Ub complex in the presence of peptides **5** and **6**. Lane 1: UBE2C-Ub complex; Lane 2: UBE2C-Ub* complex control; Lane 3: UBE2C-Ub complex in presence of 1 mM peptide **6**, used as negative control; Lane 4: UBE2C-Ub complex in presence of 25µMpeptide **5**; Lane 5: UBE2C-Ub complex in presence of 250 µM peptide **5**; Lane 6: UBE2C-Ub complex in presence of 1 mM of **5**. *Control is obtained by preparing the reaction mixture without ATP-Mg^2+^, to confirm the dependence of the ubiquitylation reaction on energy input.

To assess the functional impact of the lead peptide **5** on UBE2C activity, an *in vitro* ubiquitination assay was performed by monitoring the formation of the UBE2C-Ub thioester complex in the presence of increasing concentrations of **5** and compared to the negative control peptide **6**. The assay was designed to evaluate whether **5** interferes with the conjugation step of ubiquitin to the E2 enzyme, thereby acting as a potential inhibitor of its catalytic function. As shown in Figure 10, the formation of the UBE2C-Ub complex (∼25 kDa) decreased progressively as the concentration of **5** increased from 25 μM to 1 mM, indicating a dose-dependent inhibition. In contrast, peptide **6**, tested at 1 mM, had no apparent effect on complex formation, as the intensity of the UBE2C-Ub band remained comparable to the control condition. These findings support the biological activity of **5** as a modulator of UBE2C-dependent ubiquitin transfer.

### Serum stability of peptide 5

To further assess the biological properties of peptide **5** in cell-based assays, serum stability experiments were performed. The results are shown in Supplementary Material, Table S3. Data obtained by LC-MS analysis (data not shown) revealed that at incubation time t = 0 with serum, the entire peptide was present; after 6h incubation, the degradation profile indicates the presence of a further peak attributable to the **5** sequence lacking the first amino acid (W residue) at the N-terminus. On the basis of the docking results on the binding mode of peptides **2** and **5**, we can estimate that the loss of W^1038^ would lower the affinity but not abolish it. Indeed, the docking of **2** showed that the L^1038^ is not involved in the binding. Therefore, we can speculate that the loss of a single amino acid should not actually compromise the activity of the peptide. The percentage of potentially active peptide still present can be considered the sum of the **5** and its deleted derivative (here indicated as X), which corresponds to 70% after 6h and 52% after 24h. After 48h, only X species were present in approximately the same percentage, which halved after 72h. This result should be considered appreciable for undertaking cell-based assays.

## Discussion

UBE2C is a member of the ubiquitin conjugating enzyme family, often overexpressed in various cancers, including ovarian, colorectal, and breast cancers. Targeting this enzyme could help to inhibit cancer cell division and inhibitors of UBE2C could serve as novel chemotherapeutic agents to prevent tumour growth^^62^^.

The potential of UBE2C inhibition as a therapeutic strategy is substantiated by numerous molecular biology studies. In particular, UBE2C silencing and gene knockdown experiments in cancer models have consistently demonstrated its pivotal role in tumour progression and cell cycle regulation, thus validating this enzyme as a valuable drug target. However, despite its therapeutic relevance, to date, neither small molecules nor peptide-based inhibitors have been experimentally shown to inhibit UBE2C enzymatic activity. Indeed, very few inhibitors have been identified for E2 ubiquitin-conjugating enzymes in general, underscoring the unmet need for therapeutic innovation in this protein family.

In this context, the identification of short peptides able to modulate UBE2C activity represents a novel and potentially valuable contribution. Compared to small molecules, peptides can provide improved binding surface complementarity and can be rationally optimised for enhanced affinity and conformational behaviour. This approach is particularly well-suited to targeting UBE2C, which, like most E2 ubiquitin-conjugating enzymes, lacks defined binding pockets and presents a relatively flat surface architecture, making it extremely challenging for small molecules to achieve effective binding, thereby supporting the rationale for peptide-based inhibitors as a viable and promising strategy. UBE2C peptide inhibitors can be *ad hoc* designed to directly block the enzyme’s interaction with substrates or partner proteins (such as the E3 ligase complex), ensuring a highly targeted effect.

In this study, starting from previously published data on a peptide targeting the E1–E2 interface (**U1**), we implemented a rational design strategy grounded on key structural and functional principles. The design was guided by structural and biophysical features of U1, previously characterised as a lead scaffold for UBE2C targeting. We introduced specific sequence modifications aiming to improve physical-chemical and binding properties of **U1** derivatives. In particular, cysteines were substituted with serines to enhance stability, aromatic residues were introduced to improve quantification through UV absorbance, and the role of negatively charged residues in the interaction was evaluated. We found that substituting cysteines with serines preserved activity, while introducing a tryptophan residue at the N-terminal position increased activity, indicating that this residue can be accommodated without negatively impacting interaction with UBE2C. Our results also demonstrated the critical functional importance of acidic residues D^1042^, E^1046^, and D^1047^, whose substitution resulted in complete activity loss. Conversely, the partial retention of activity upon mutation of E^1043^ suggested that this residue is less critical for binding, indicating a potential site for future optimisation. Collectively, these findings led us to select peptide 5 as the reference compound for further experimental validation.

To reinforce the binding affinity data of peptide **5**, we combined MST and AlphaScreen assays, leveraging two distinct detection principles (thermophoresis and proximity-based energy transfer), thus increasing the robustness of our experimental validation.

We used PT-WTE simulations to explore the conformational landscape of U1-derived peptides in aqueous solution. PT-WTE, an enhanced sampling technique particularly suited to small peptides, allowed efficient exploration of rare conformational states and metastable intermediates typically inaccessible to conventional simulations. All peptides shared a global minimum corresponding to a disordered state but differed significantly in their ability to access folded conformations. Peptides U1 and **5**, showing either a well-defined (U1) or dynamically accessible (**5**) β-sheet-like folded basin, exhibited comparable binding affinities. In contrast, peptide **2** presented a significantly higher energy barrier to reach the folded state, aligning with its reduced activity (∼3-fold lower than **U1** and **5**). Peptide **6**, although similar to **5** in the unfolded region, lacked stabilised intermediate or folded conformations and was completely inactive, likely due to both conformational and electrostatic effects from the E1046Y mutation.

Docking calculations, performed using representative conformations from PT-WTE simulations, further provided structural insights into peptide-UBE2C interactions. For peptide **5**, docking revealed interactions involving both electrostatic and hydrophobic contacts, notably with residues K^33^, Q^36^, Q^37^, and T^41^ of UBE2C. The introduced L1038W mutation contributed additional hydrophobic contacts, resulting in enhanced binding affinity compared to peptide **2**. The stability of the peptide–protein complex observed during 200 ns of molecular dynamics simulations corroborates the docking results. The persistence of key interactions in the H1 region confirms the reliability of the predicted binding mode. MM/PBSA calculations confirm the energetic stability of the complex, with a highly favourable ΔG_bind_ (–359.31 kJ/mol) driven primarily by electrostatics and supported by van der Waals and hydrophobic contributions. These findings support a consistent and robust peptide–UBE2C interface over time. Moreover, the conformation of peptide in the resulting binding mode results align with PT-WTE simulations and experimental binding data, suggesting that peptide **5** adopts a partially ordered conformation conducive to target engagement, possibly through a fly-casting mechanism. Docking outcomes were consistent with SAR data, emphasising residues D^1047^, E^1046^, and E^1049^ as critical for binding, while confirming the non-essential role of E^1043^.

CD analysis confirmed that, despite local differences in secondary structure and folding pathways identified computationally, peptides predominantly remained disordered in solution. This supported the hypothesis that structured conformations observed computationally may form upon binding to UBE2C, reinforcing the potential fly-casting mechanism suggested by simulations.

Considering proteolytic instability as a critical limitation for peptides, peptide **5** was evaluated for serum stability. Remarkably, peptide **5** displayed significant stability, with approximately 50% remaining intact after 24 h, further underscoring its suitability for biological applications.

All data collected herein position peptide **5** as a promising inhibitor of UBE2C, highlighting the need for future cellular assays to confirm its therapeutic potential^25^.

## Supplementary Material

Supplementary Material clean copy anonymous.docx

## Data Availability

The data that support the findings of this study are available from the corresponding authors upon request.
