# Supplementary material for: Identification of the first peptide inhibitor of UBE2C enzymatic activity: insights from metadynamics-guided folding and binding studies
Source: J Enzyme Inhib Med Chem. 2026 Jan 6;41(1):2605383. doi: 10.1080/14756366.2025.2605383 (PMC12777749; doi:10.1080/14756366.2025.2605383)
Supplement: Supplementary Material clean copy anonymous.docx [file IENZ_A_2605383_SM1434.docx]

**Supplementary Material for**

**Identification of the first peptide inhibitor of UBE2C enzymatic activity: Insights from Metadynamics-Guided Folding and Binding Studies**

**Table of Contents**

**SI Supplemental Figures and Figure Legends**

**Figure S1:** Design approach of the lead **U1** and **L2** peptides from the work of Correale et al. [25]

**Figure S2:** Ramachandran plot of the peptides **U1**, **2**, **5** and **6** conformations retrieved from PT-WTE.

**Figure S3:** 2D-FES time evolution during the 250 ns of PT-WTE of **U1**.

**Figure S4:** 2D-FES time evolution during the 250 ns of PT-WTE of peptide **2**.

**Figure S5:** 2D-FES time evolution during the 250 ns of PT-WTE of peptide **5**.

**Figure S6:** 2D-FES time evolution during the 250 ns of PT-WTE of peptide **6**.

**Figure S7:** Replica exchange plots during the PT-WTE simulation of peptide **U1**.

**Figure S8:** Replica exchange plots during the PT-WTE simulation of peptide **2**.

**Figure S9**. Replica exchange plots during the PT-WTE simulation of peptide **5**.

**Figure S10**: Replica exchange plots during the PT-WTE simulation of peptide **6**.

**Figure S11:** Reweighted 2D-FES of **U1.**

**Figure S12:** Superposition between the X-ray conformation of **U1** (U1_xr_) and that of basin C from PT-WTE.

**Figure S13:** in vitro ubiquitylation assay

**Figure S14:** Microscale thermophoresis (MST) for **U1** derivates peptides *versus* UBE2C*.*

**Figure S15:** 1D-FES of peptides **U1** and **2**.

**Figure S16:** Far UV-CD of peptides **2**, **5**, and **6**.

**Figure S17.** Average RMSD plot calculated on the backbone atoms of A) UbcH10 and B) peptide **5.**

**Figure S18.** Cluster analysis of 200 ns MDs of peptide **5** in the UbcH10.

**Supplemental Tables**

**Table S1:** Docking and refinement results from HADDOCK for the three peptide–protein complexes.

**Table S2:** PRODIGY ΔG predictions of each refined complex obtained from HADDOCK.

**Table S3**: Serum stability assay of peptide **5**.


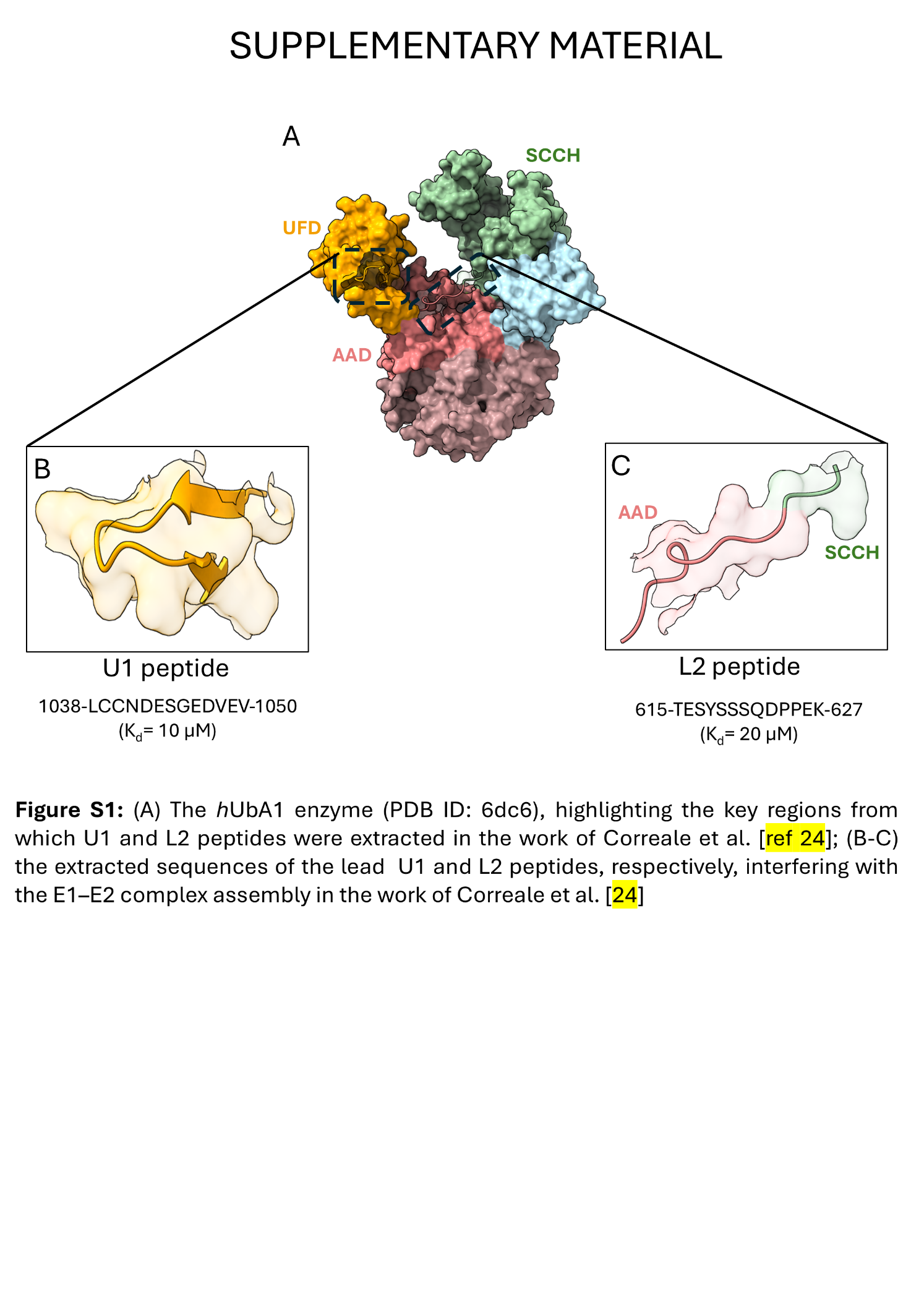


**Figure S1:** (A) The hUbA1 enzyme (PDB ID: 6DC6), highlighting the key regions from which U1 and L2 peptides were extracted in the work of Correale et al. [25]; (B-C) the extracted sequences of the lead **U1** and **L2** peptides, respectively, interfering with the E1–E2 complex assembly in the work of Correale et al. [25]


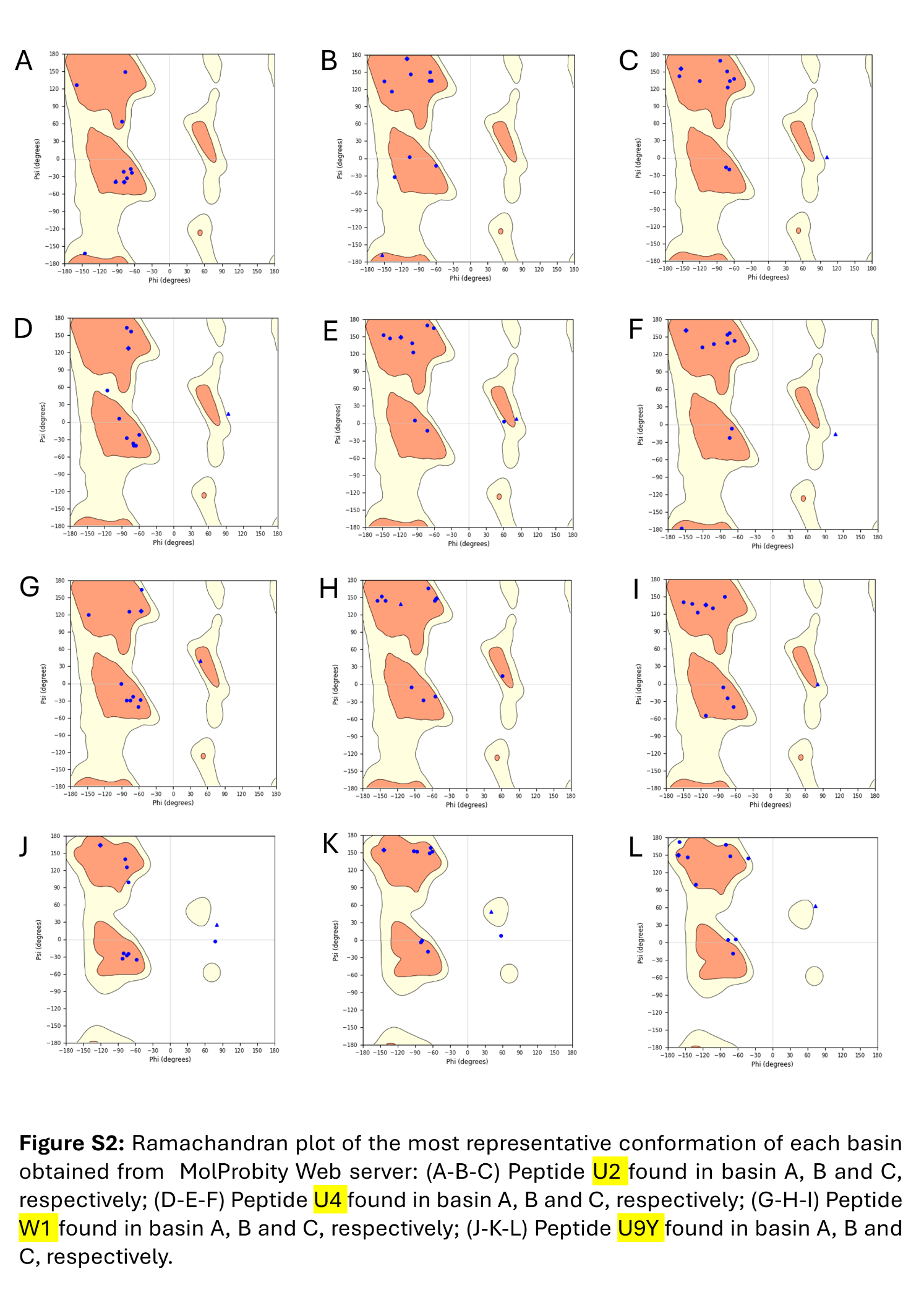


**Figure S2:** Ramachandran plot of the most representative conformation of each basin obtained from Maestro GUI: (A-B-C) Peptide **U1** found in basin A, B and C, respectively; (D-E-F) Peptide **2** found in basin A, B and C, respectively; (G-H-I) Peptide **5** found in basin A, B and C, respectively; (J-K-L) Peptide **6** found in basin A, B and C, respectively.


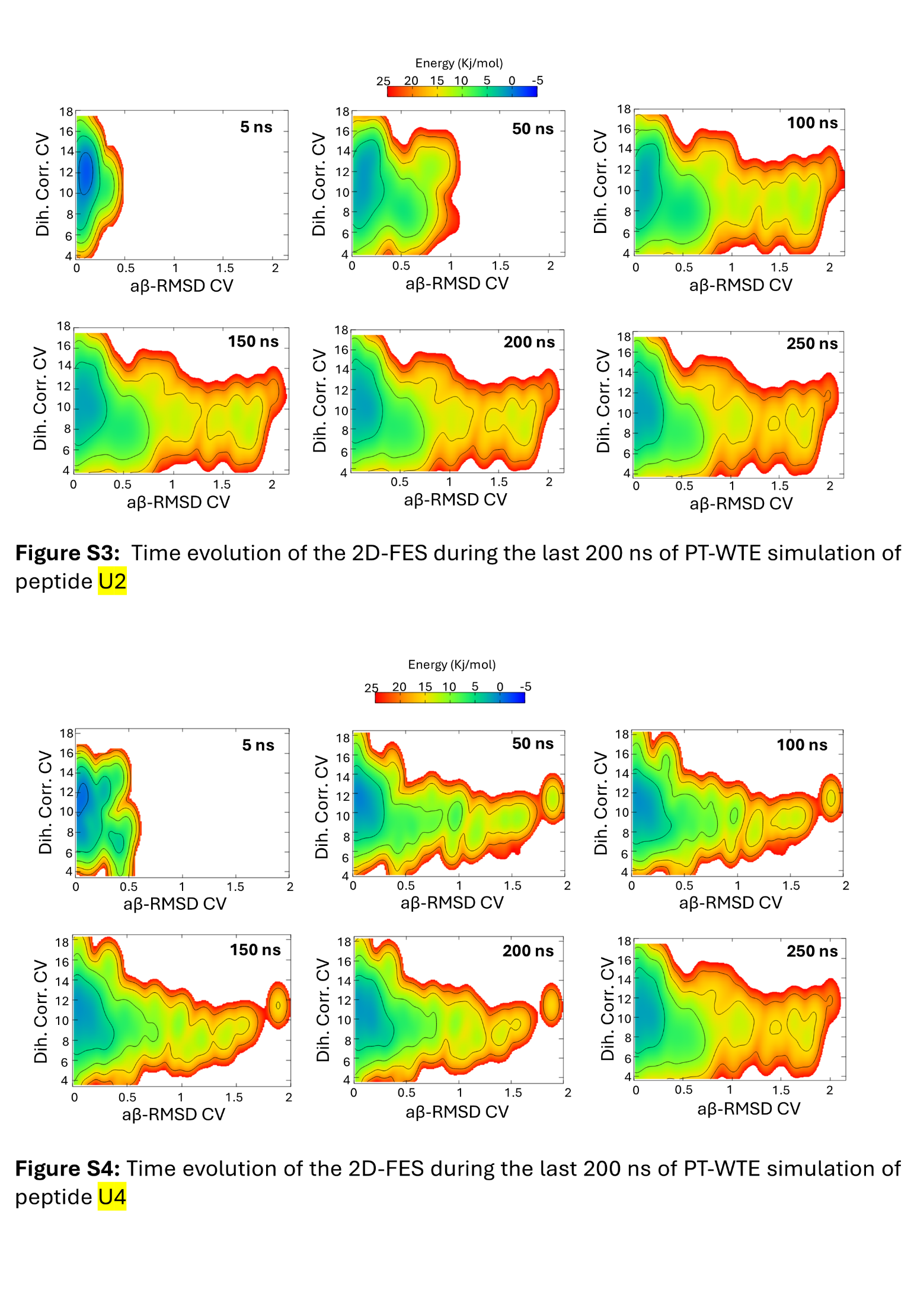


**Figure S3:** Time evolution of the 2D-FES during the 250 ns of PT-WTE simulation of the lead peptide **U1**.


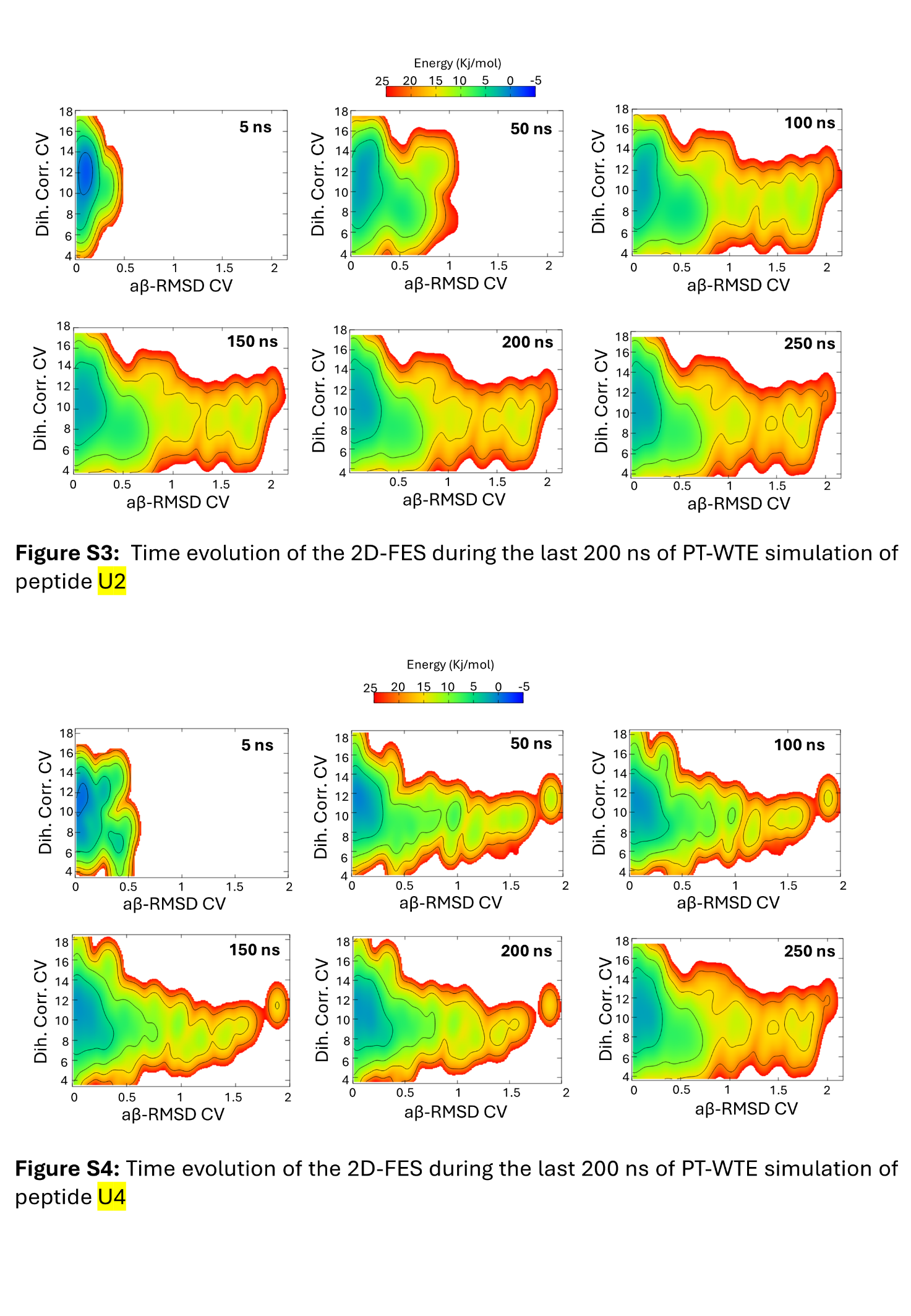


**Figure S4:** Time evolution of the 2D-FES during the 250 ns of PT-WTE simulation of peptide **2**.

**
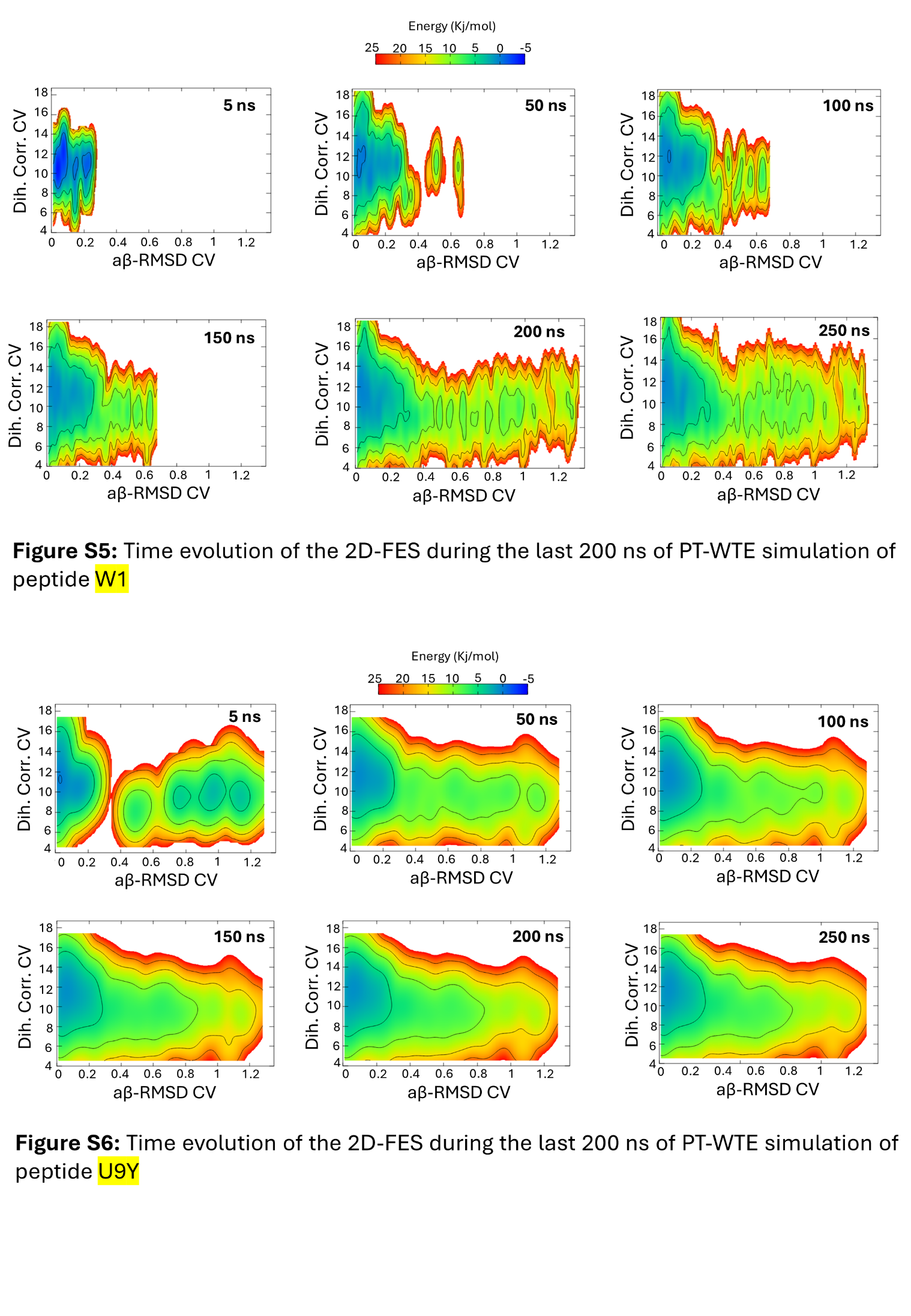
**

**Figure S5:** Time evolution of the 2D-FES during the 250 ns of PT-WTE simulation of peptide **5**.

**
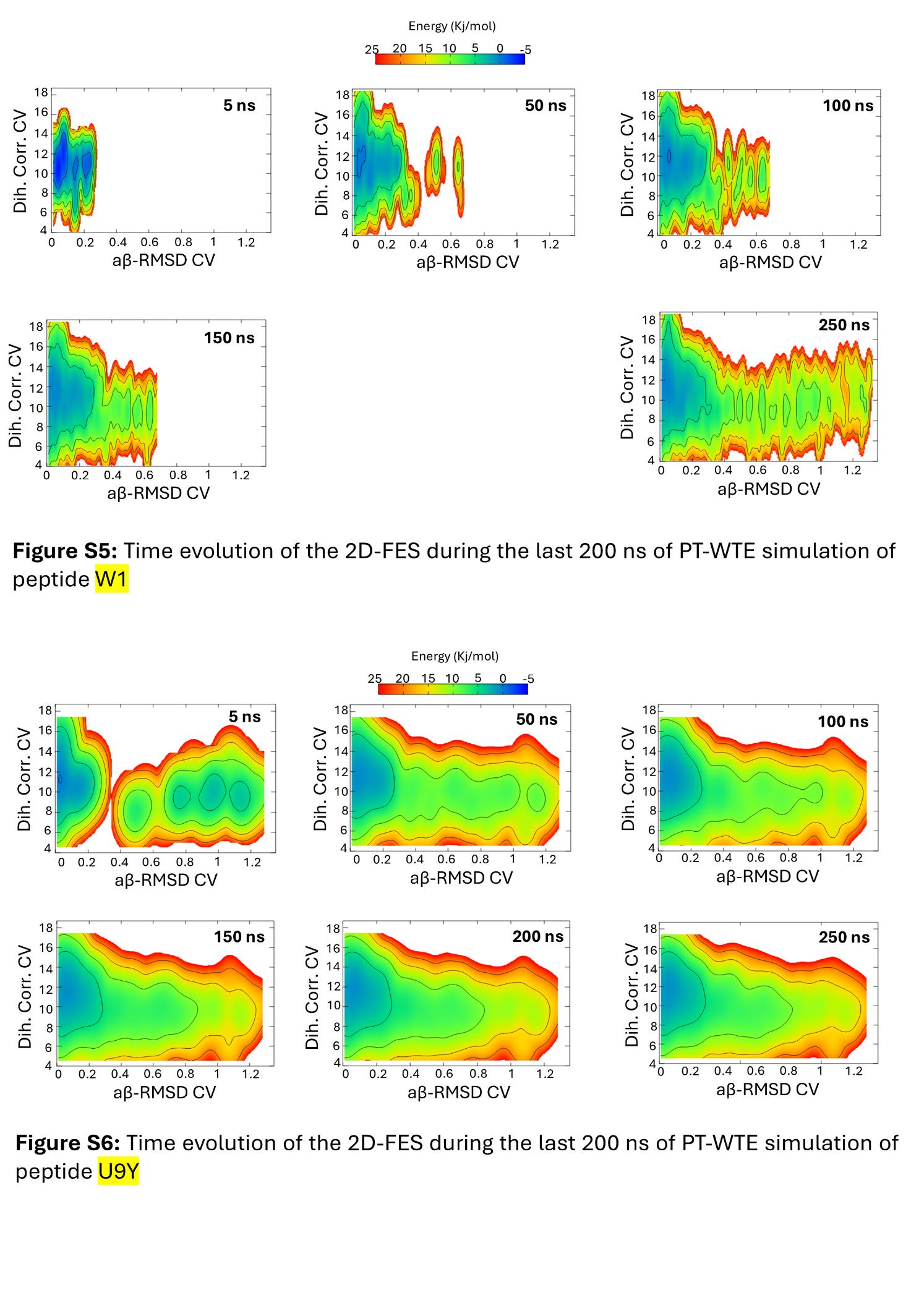
**

**Figure S6:** Time evolution of the 2D-FES during the 250 ns of PT-WTE simulation of peptide **6**.


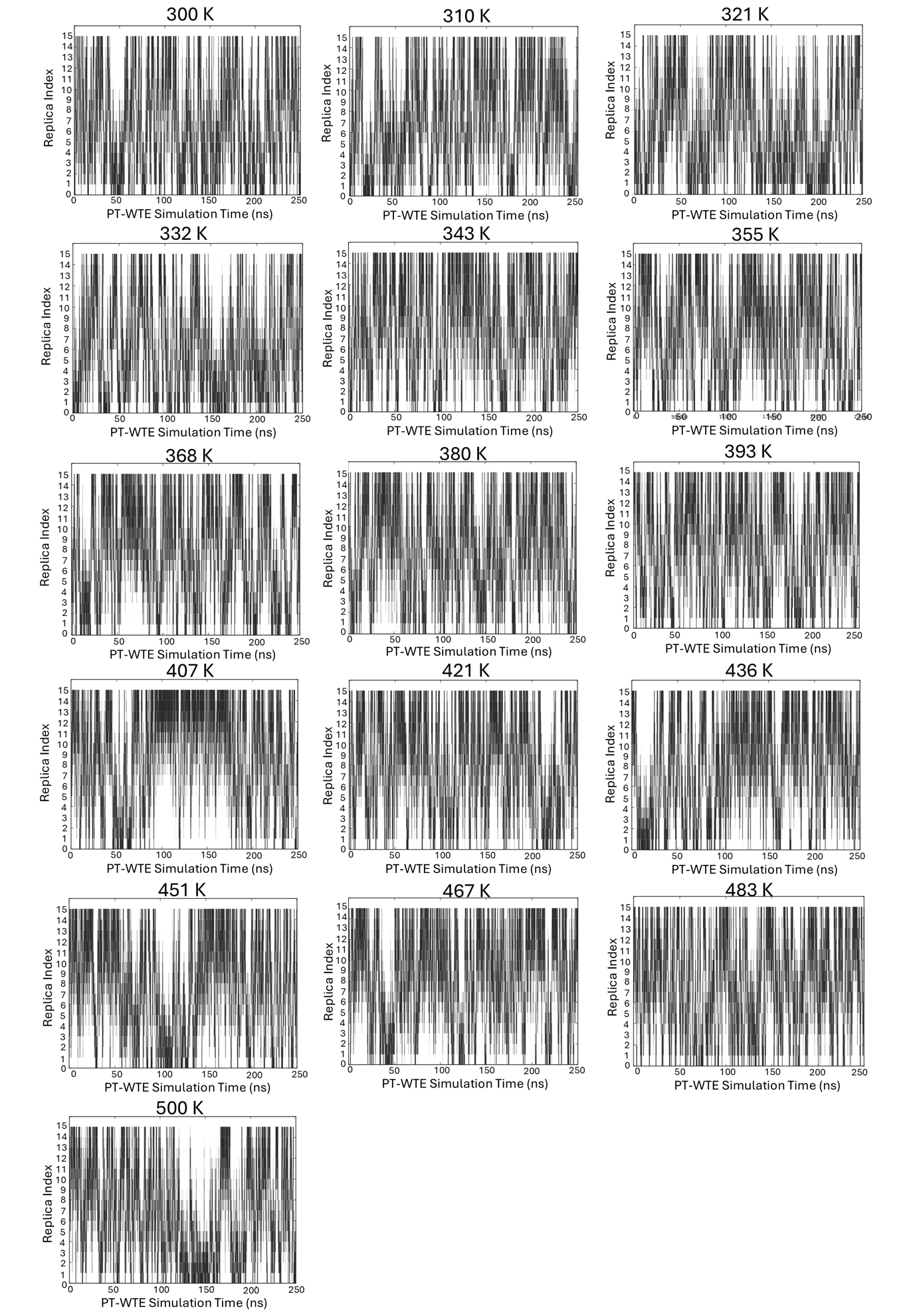


**Figure S7:** Replica exchange plots during the PT-WTE simulation of peptide **U1.**

**
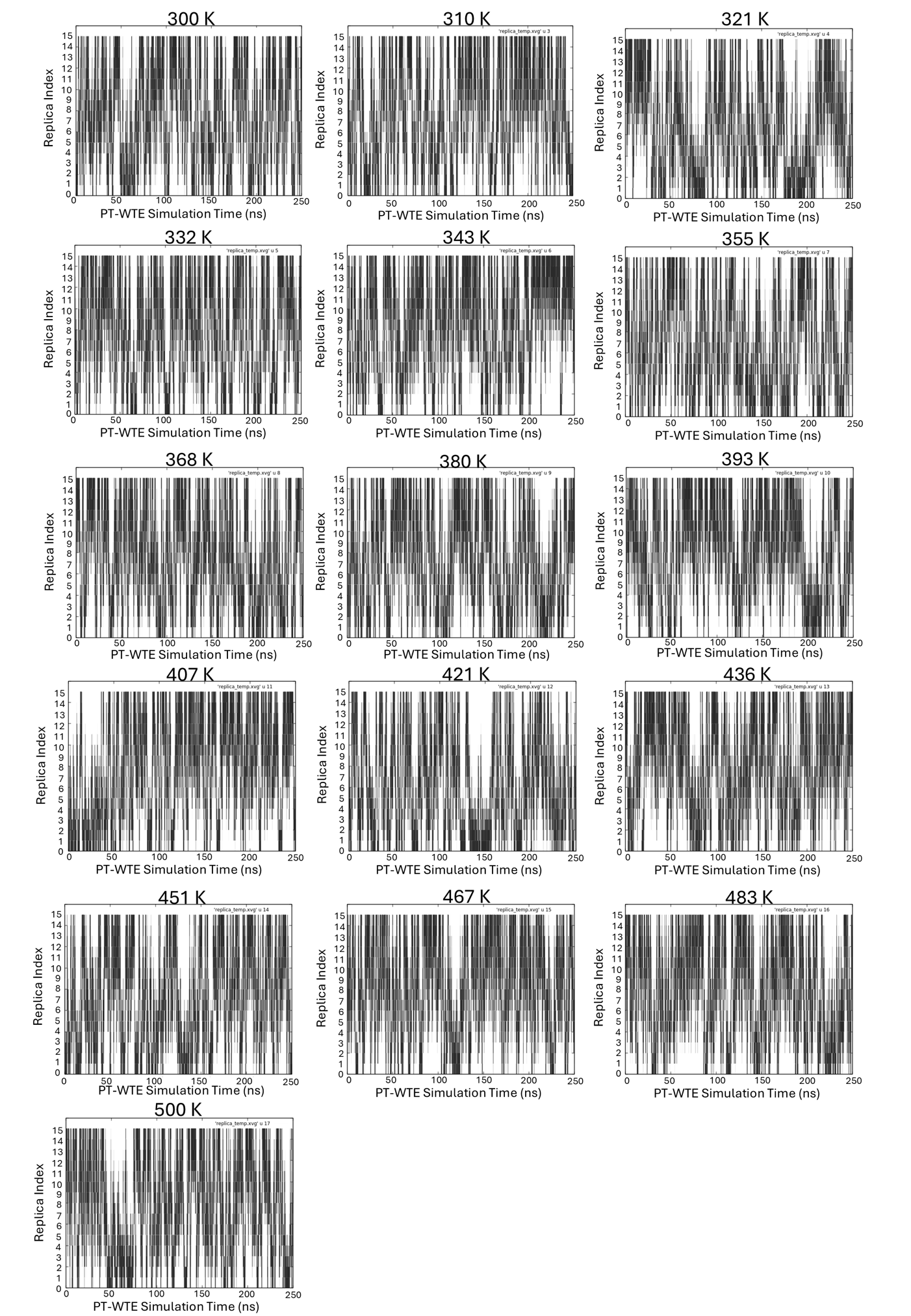
**

**Figure S8:** Replica exchange plots during the PT-WTE simulation of peptide **2**.


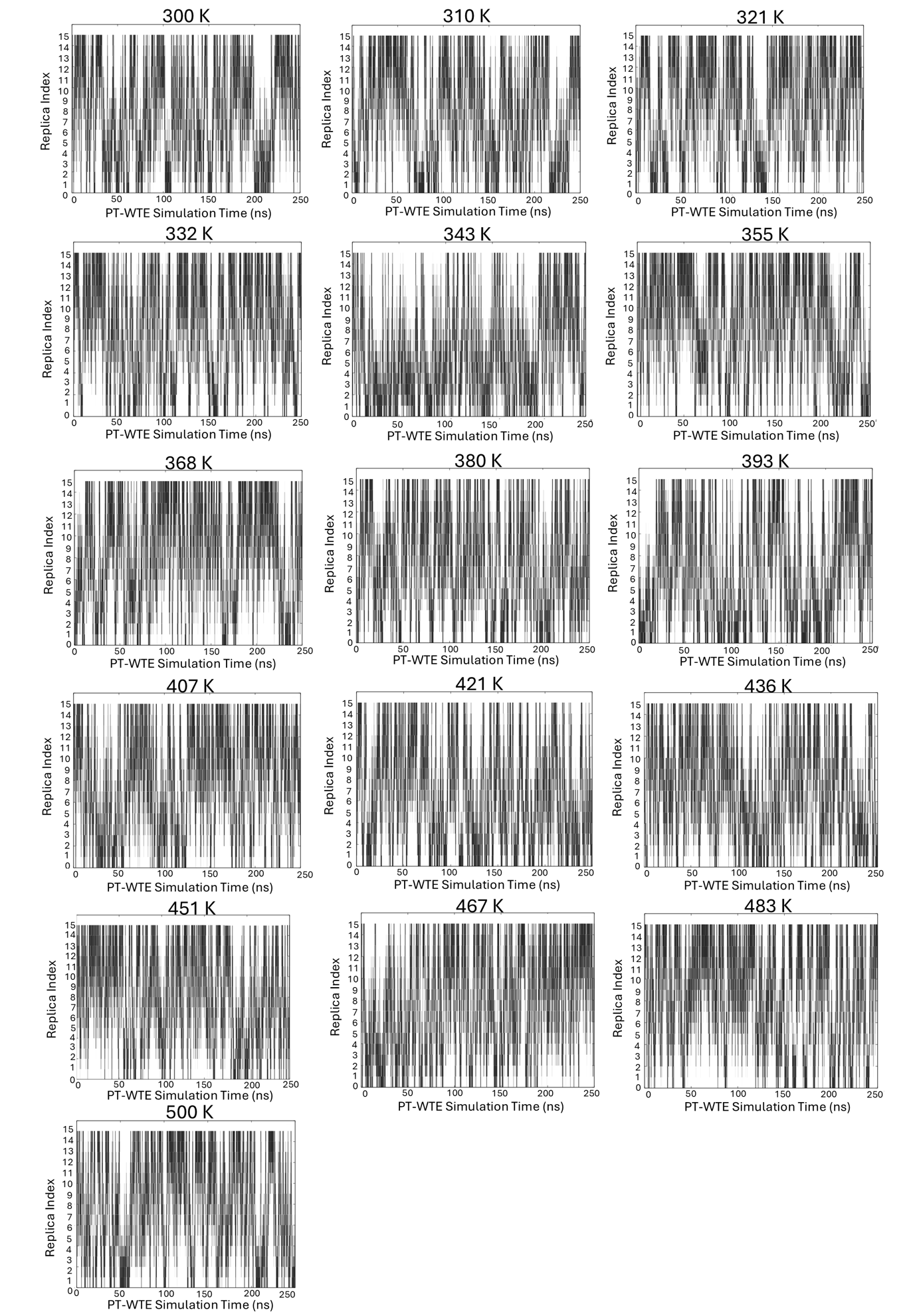


**Figure S9:** Replica exchange plots during the PT-WTE simulation of peptide **5**.


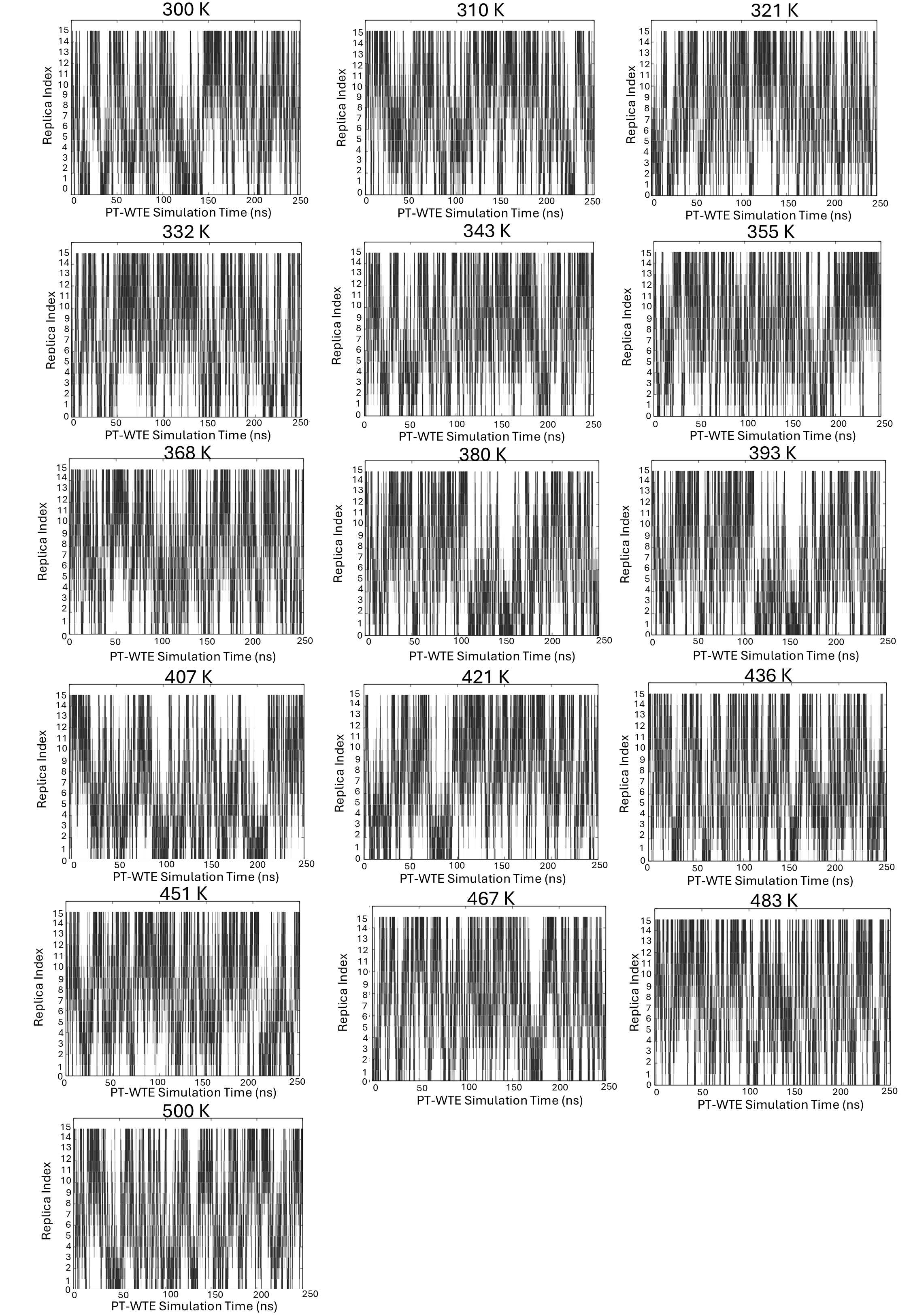


**Figure S10:** Replica exchange plots during the PT-WTE simulation of peptide **6**.


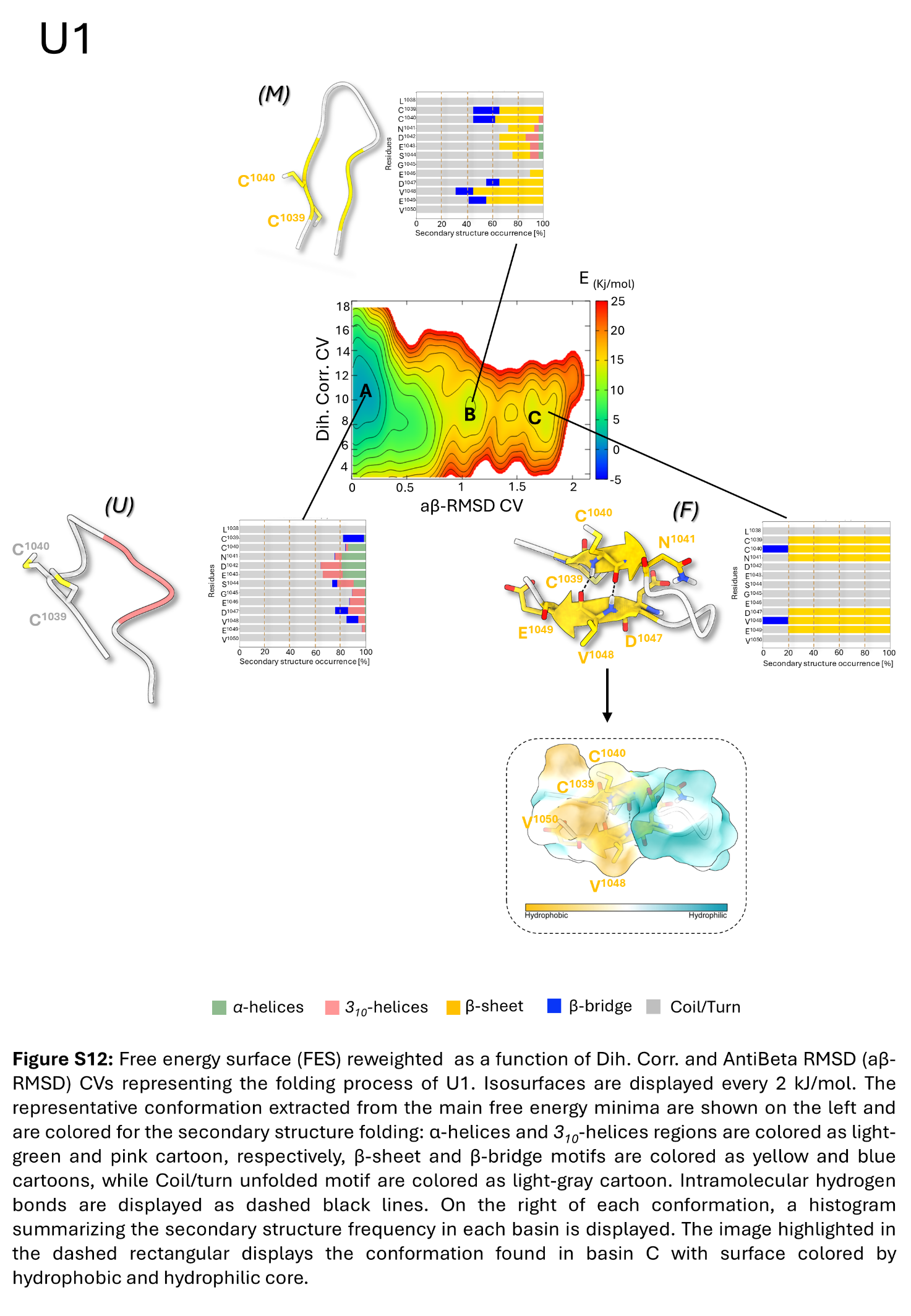


**Figure S11:** Free energy surface (FES) reweighted as a function of Dih. Corr. and AntiBeta RMSD (aβ-RMSD) CVs representing the folding process of **U1**. Isosurfaces are displayed every 2 kJ/mol. The representative conformation extracted from the main free energy minima are shown on the left and are colored for the secondary structure folding: α-helices and *3_10_*-helices regions are colored as light-green and pink cartoon, respectively, β-sheet and β-bridge motifs are colored as yellow and blue cartoons, while Coil/turn unfolded motif are colored as light-gray cartoon. Intramolecular hydrogen bonds are displayed as dashed black lines. On the right of each conformation, a histogram summarizing the secondary structure frequency in each basin is displayed. The image highlighted in the dashed rectangular displays the conformation found in basin C with surface colored by hydrophobic and hydrophilic core.


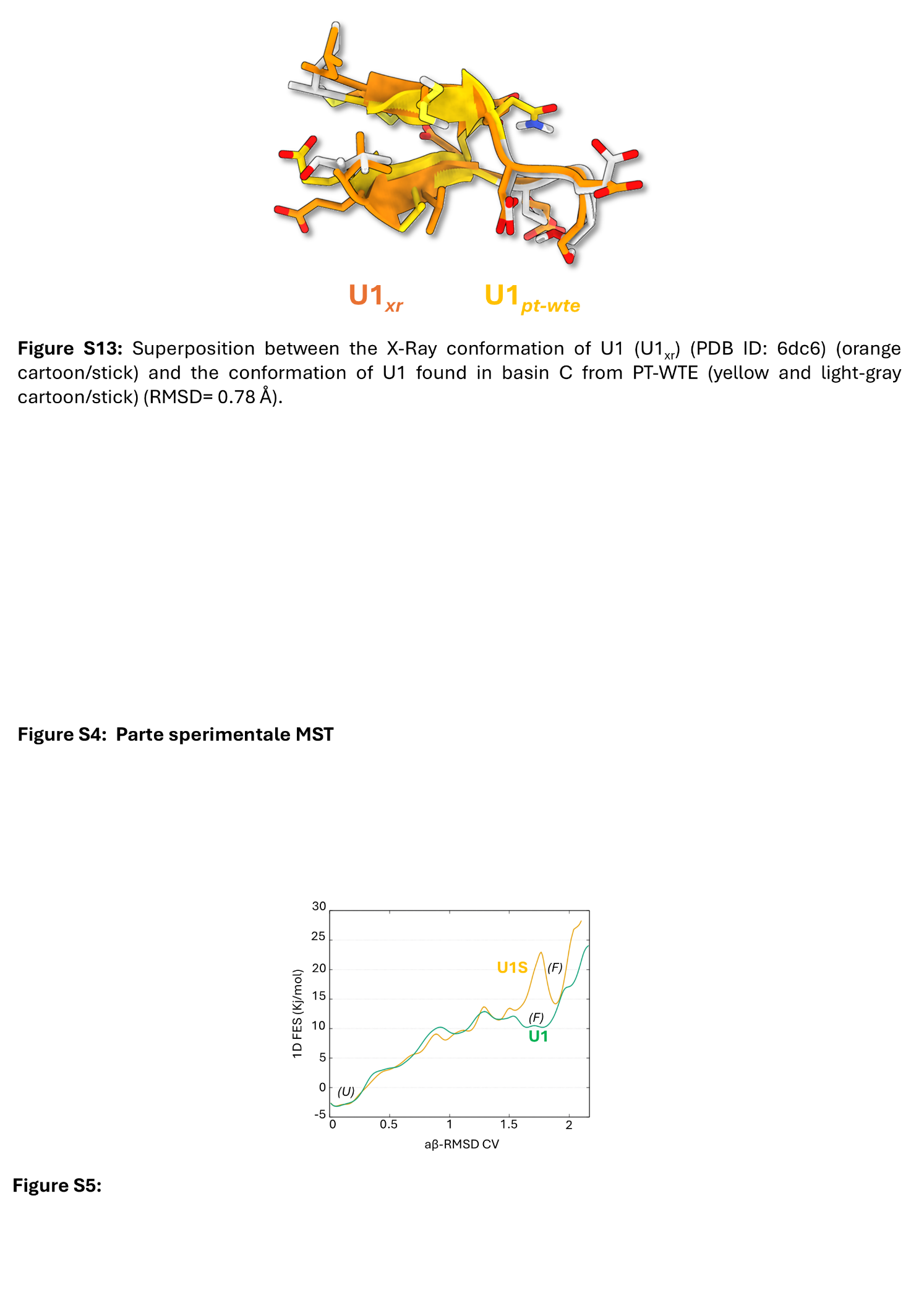


**Figure S12:** Superposition between the X-Ray conformation of U1 (U1_xr_) (PDB ID: 6DC6) (orange cartoon/stick) and the conformation of **U1** found in basin C from PT-WTE (yellow and light-gray cartoon/stick, respectively) (RMSD= 0.78 Å).


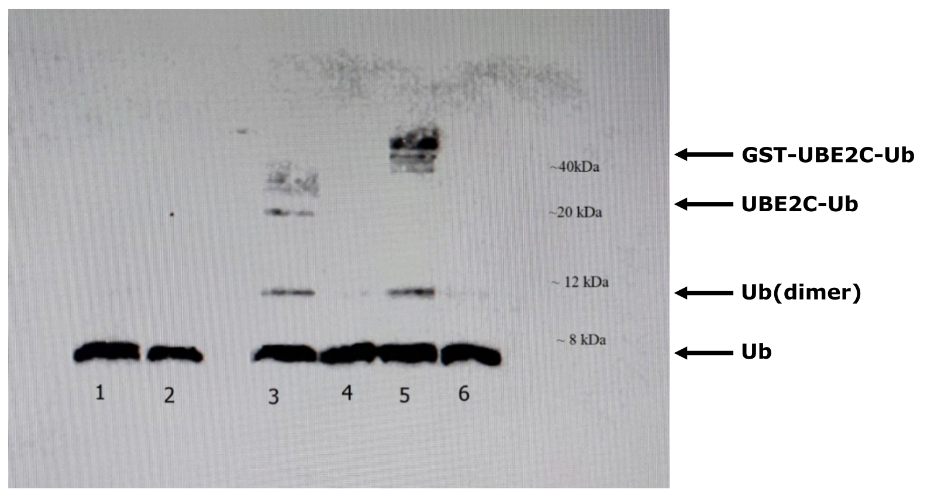


**Figure S13.** Validation of UBE2C enzymatic activity and assessment of GST-tag impact through in vitro ubiquitylation assay. In lane 1, the formation of the E1–Ub complex (~100 kDa) was expected as the first step of the ubiquitylation cascade, although the corresponding band was not visible due to incomplete transfer of high molecular weight species. Lanes 3 and 5 represent reaction mixtures containing either cleaved UBE2C (lane 3) or GST-tagged UBE2C (lane 5) as E2 enzyme. In both samples, the presence of characteristic bands for ubiquitin monomer (~8 kDa), di-ubiquitin (~12 kDa), and UBE2C-Ub thioester complexes (~25 and ~45 kDa) confirms that both protein forms are enzymatically active and capable of catalysing These results support the use of both GST–UBE2C and cleaved UBE2C as functionally competent enzymes in downstream binding and activity assays. Lane 1: Mix E1-Ub; Lane 2: E1-Ub* Complex control *; Lane 3: UBE2C-Ub Complex mix; Lane 4: UBE2C-Ub* Complex mix control; Lane 5: UBE2C-GST-Ub Complex mix; Lane 6: UBE2C-GST-Ub* Complex mix control. *The controls for each reaction consist of the respective reaction mixture in the absence of ATP-Mg^2+^.


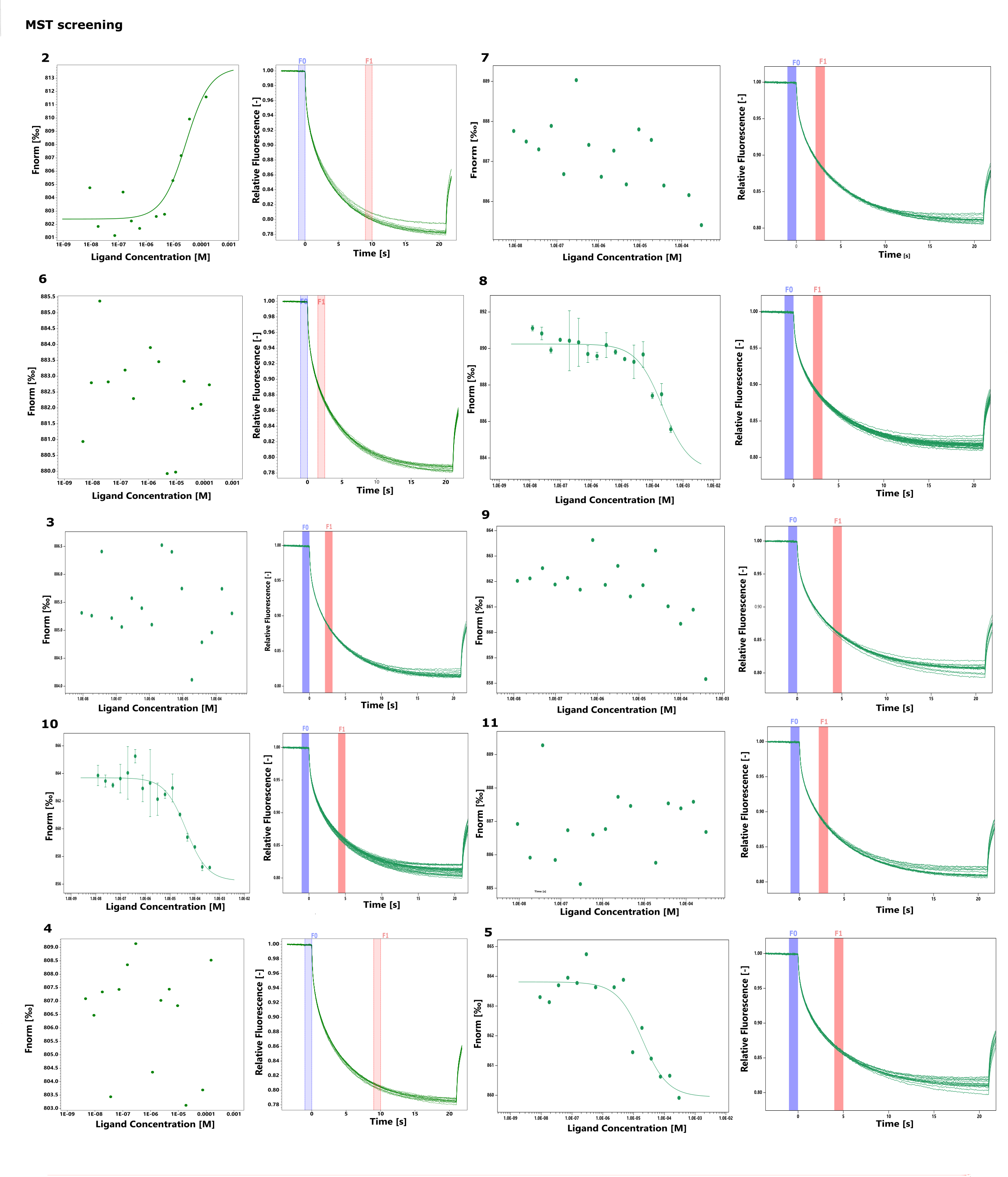


**Figure S14.** Microscale thermophoresis (MST) for **U1** derivates peptides *versus* UBE2C*.* Plot of normalized fluorescence obtained from peptides binding experiment versus UBE2C at different concentrations derived from MST experiments and the MST traces were reported.


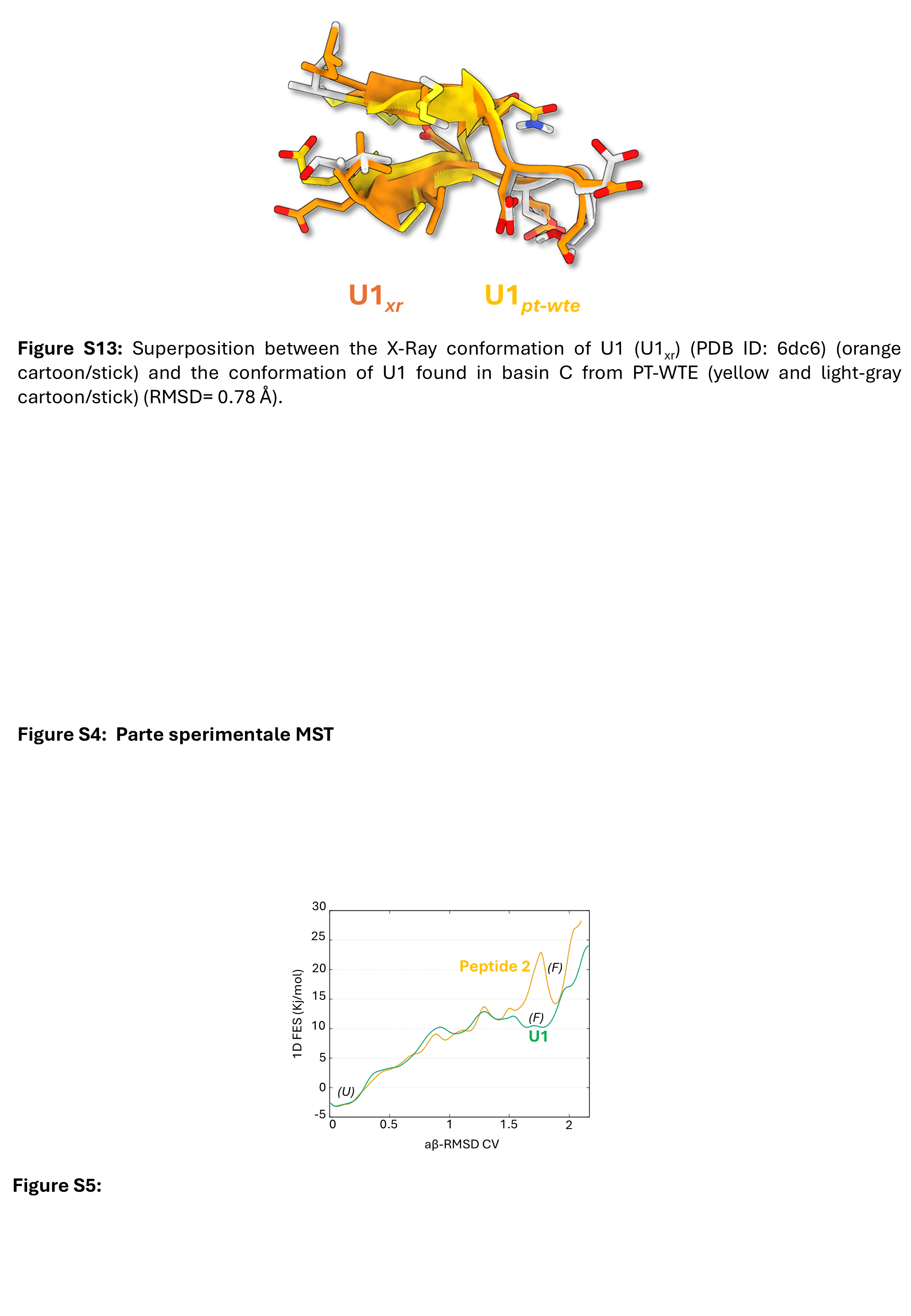


**Figure S15:** Overlap of the 1D-FES as function of aβRMSD CV of **U1** (green line) and peptide **2 (**yellow line), highlighting the different transition state between the unfolded (U) and folded (F) conformation.





**Figure S16:** Far UV-CD of peptides **2**, **5**, and **6**.

**
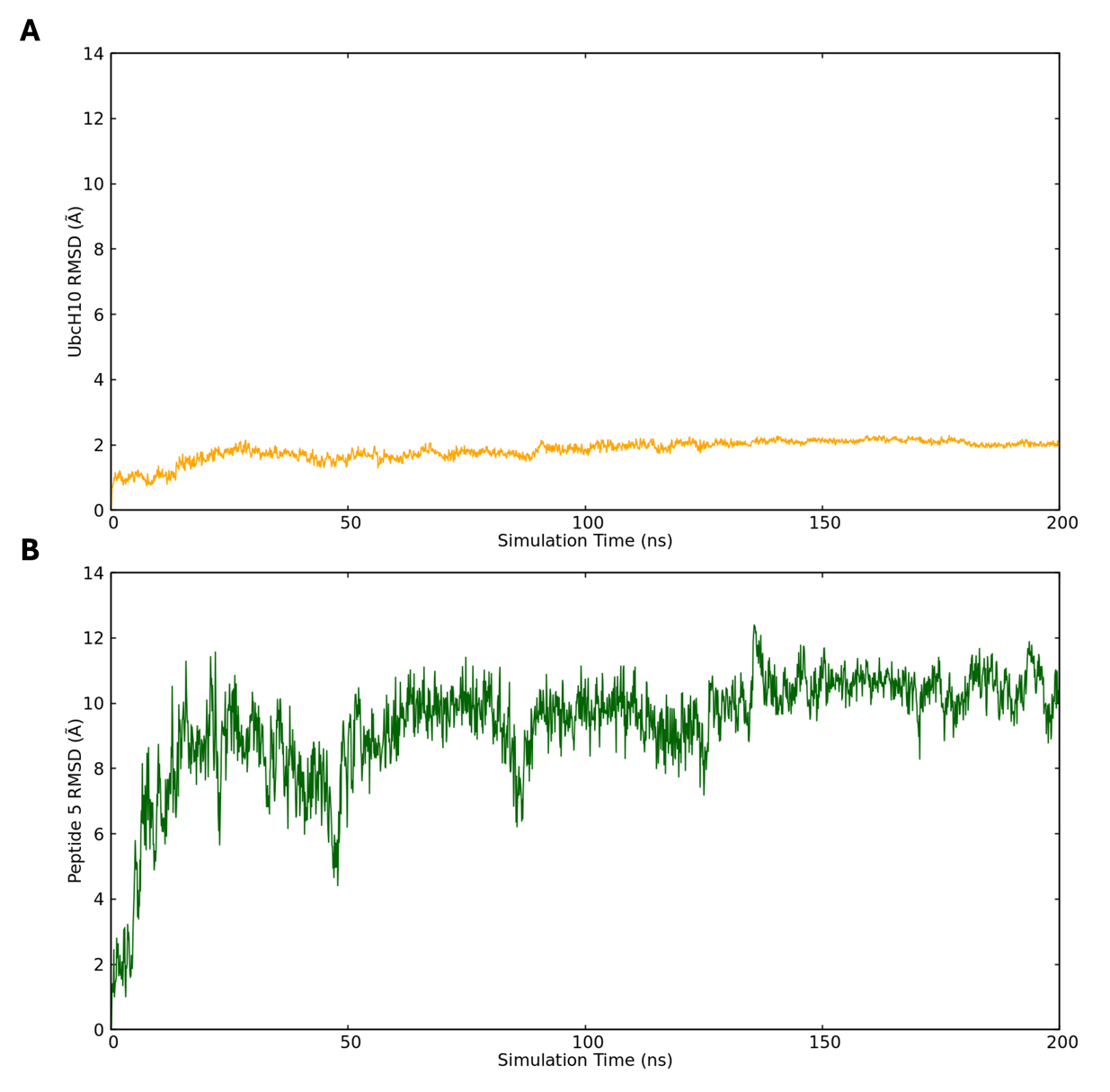
**

**Figure S17.** Average RMSD plot calculated on the backbone atoms of A) UbcH10 and B) peptide **5.**


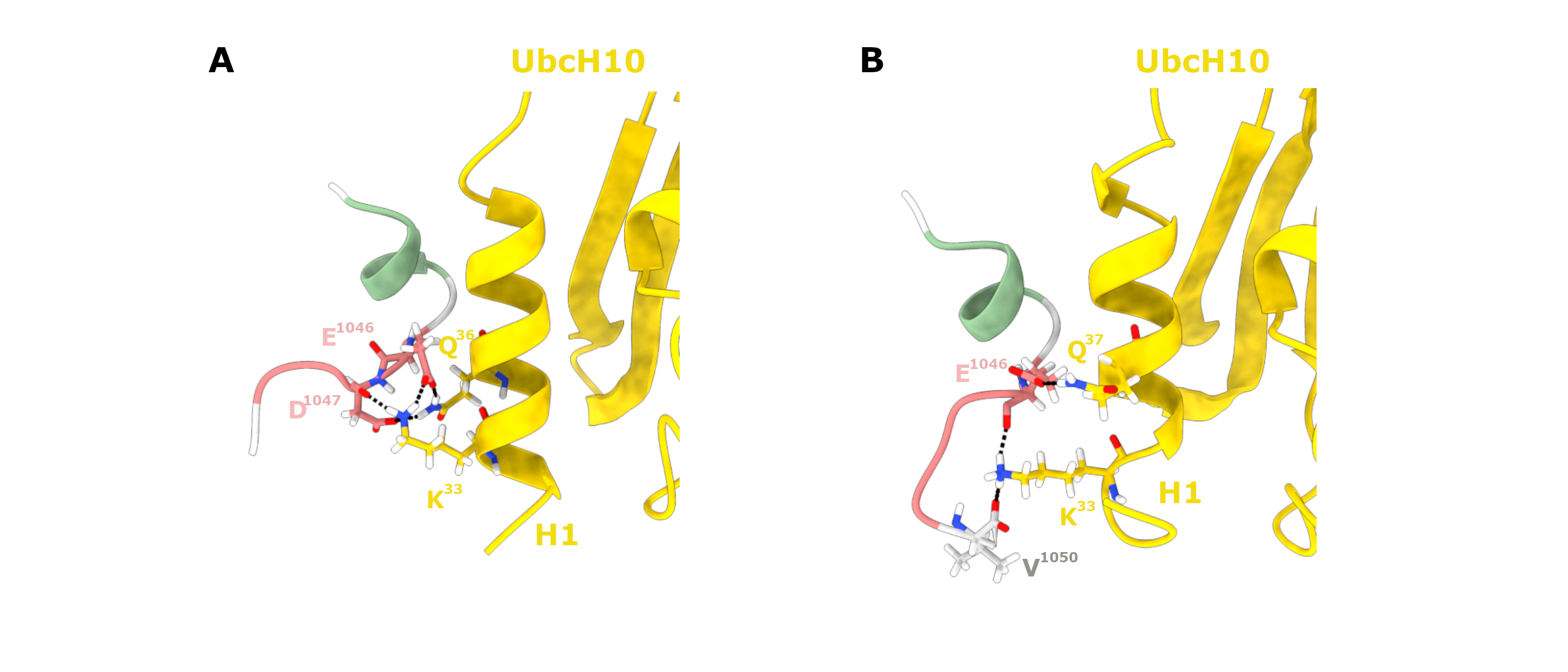


**Figure S18.** Cluster analysis of 200 ns MDs of peptide **5** in the UbcH10. The structures correspond to the centroid of A) cluster 1 and B) cluster 2 extracted from the 200-ns MD trajectory. UBE2C is shown as a yellow cartoon, while the peptide is visualized in cartoon format and colored according to its secondary structure, following the scheme used in the PT-WTE analysis: α-helices and 3₁₀-helices in light green and pink, β-sheets and β-bridges in yellow and blue, and coil/turn regions in light grey. Interacting residues are shown as sticks and labelled; hydrogen bonds are represented as black dashed lines.

| **Peptide** | **Cluster ID** | **HADDOCK Score** | **Refined Complex Score** |
| --- | --- | --- | --- |
| **2** | Cluster 1 | -38.3 +/- 0.7 | -50.4 +/- 0.7 |
| **2** | Cluster 4 | -32.5 +/- 1.4 |  |
| **2** | Cluster 3 | -28.3 +/- 0.8 |  |
| **2** | Cluster 7 | -27.2 +/- 1.9 |  |
| **2** | Cluster 5 | -26.9 +/- 2.9 |  |
| **2** | Cluster 9 | -26.7 +/- 6.8 |  |
| **2** | Cluster 2 | -26.4 +/- 1.0 |  |
| **2** | Cluster 6 | -23.9 +/- 1.9 |  |
| **2** | Cluster 12 | -23.0 +/- 5.1 |  |
| **2** | Cluster 10 | -22.2 +/- 2.5 |  |
| **5** | Cluster 2 | -49.6 +/- 0.6 | -65.4 +/- 3.1 |
| **5** | Cluster 8 | -41.4 +/- 8.0 |  |
| **5** | Cluster 4 | -40.5 +/- 2.7 |  |
| **5** | Cluster 5 | -39.5 +/- 1.4 |  |
| **5** | Cluster 3 | -37.9 +/- 1.5 |  |
| **5** | Cluster 1 | -32.9 +/- 0.8 |  |
| **5** | Cluster 12 | -31.6 +/- 4.6 |  |
| **5** | Cluster 13 | -31.5 +/- 2.7 |  |
| **5** | Cluster 11 | -31.2 +/- 7.3 |  |
| **5** | Cluster 10 | -31.2 +/- 3.3 |  |
| **6** | Cluster 1 | -37.8 +/- 1.0 | -43.0 +/- 0.6 |
| **6** | Cluster 4 | -28.4 +/- 3.6 |  |
| **6** | Cluster 10 | -27.9 +/- 4.6 |  |
| **6** | Cluster 2 | -27.9 +/- 3.0 |  |
| **6** | Cluster 9 | -26.8 +/- 4.9 |  |
| **6** | Cluster 3 | -24.8 +/- 3.0 |  |
| **6** | Cluster 8 | -24.6 +/- 3.2 |  |
| **6** | Cluster 7 | -24.2 +/- 2.2 |  |
| **6** | Cluster 11 | -22.7 +/- 5.1 |  |
| **6** | Cluster 6 | -21.9 +/- 2.2 |  |

**Table S1**. Docking and refinement results for the three peptide–protein complexes. For each complex, the top 10 clusters obtained from HADDOCK docking are reported along with their corresponding HADDOCK scores (mean ± standard deviation) and rank based on score. The best-scoring complex for each peptide was further refined using HADDOCK refinement protocol, and the corresponding refined HADDOCK score is shown in the last column.

| **Refined Complex** | **ΔG**  **(kcal mol-1)** | **K*_d_*(M) at 25 ℃** | **ICs charged-charged** | **ICs charged-polar** | **ICs charged-apolar** | **ICs polar-polar** | **ICs polar-apolar** | **ICs apolar-apolar** | **NIS charged** | **NIS apolar** |
| --- | --- | --- | --- | --- | --- | --- | --- | --- | --- | --- |
| **2** | -6.4 | 1.9e^-05^ | 2 | 5 | 5 | 2 | 4 | 2 | 24.44 | 39.26 |
| **5** | -7.0 | 7.5e^-06^ | 3 | 5 | 4 | 4 | 8 | 2 | 25.0 | 38.64 |
| **6** | -6.2 | 2.6e^-05^ | 1 | 3 | 4 | 0 | 3 | 3 | 22.76 | 41.38 |

**Table S2**. PRODIGY results for each refined complex. Values for the Gibbs free energy change (ΔG) and the dissociation constant (Kd​) are reported, which PRODIGY predicts as indicators of the complex's thermodynamic stability and binding affinity. The "ICs" (Interfacial Contacts) columns quantify the various interaction types (charged, polar and apolar) present at the binding interface, while the "NIS" (Non-Interfacial Surface) metrics describe the composition (charged and apolar) of the complex surface not involved in the contact. Collectively, these metrics contribute to characterize the binding interface and explain the observed affinities.

| **TIME (h)** | **0** | **6** | **24** | **48** | **72** |
| --- | --- | --- | --- | --- | --- |
|  |  |  |  |  |  |
| **%** | 98 (peptide **5**) | 70 (peptide **5** + X) | 52 (peptide **5** + X) | 57 (X) | 26 (X) |
|  |  |  |  |  |  |
|  |  |  | X = peptide 5 deleted |  |  |

**Table S3**. Serum stability results obtained after incubation of peptide **5** at 37 °C for 72 h. Samples were collected at different times and analyzed by LC-MS (data not shown); data were reported as percentage of intact peptide **5** and sum of peptide **5** and its deleted derivative (X) at 0, 6, 24, 48 and 72 h
